# DSA-DeepFM: a dual-stage attention-enhanced DeepFM model for predicting anticancer synergistic drug combinations

**DOI:** 10.1093/bioadv/vbaf269

**Published:** 2025-10-27

**Authors:** Yuexi Gu, Yongheng Sun, Louxin Zhang, Jian Zu

**Affiliations:** School of Mathematics and Statistics, Xi’an Jiaotong University, Shaanxi 710049, People’s Republic of China; School of Mathematics and Statistics, Xi’an Jiaotong University, Shaanxi 710049, People’s Republic of China; Department of Mathematics and Centre for Data Science and Machine Learning, National University of Singapore, Singapore 119076, Singapore; School of Mathematics and Statistics, Xi’an Jiaotong University, Shaanxi 710049, People’s Republic of China

## Abstract

**Motivation:**

Drug combinations are crucial in combating drug resistance, reducing toxicity, and improving therapeutic outcomes in disease management. Because a large number of drugs are available, the potential combinations increase exponentially, making it impractical to rely solely on biological experiments to identify synergistic combinations. Consequently, machine learning methods are increasingly being used to find synergistic drug combinations. Most existing methods focus on predictive performance through auxiliary data or complex models, but neglecting underlying biological mechanisms limits their accuracy in predicting synergistic drug combinations.

**Results:**

We present DSA-DeepFM, a deep learning model that integrates a dual-stage attention (DSA) mechanism with Factorization Machines (FMs) to predict synergistic two-drug combinations by addressing complex biological feature interactions. The model incorporates categorical and auxiliary numerical inputs to capture both field-aware and embedding-aware patterns. These patterns are then processed by a deep FM module, which captures low- and high-order feature interactions before making the final predictions. Validation testing demonstrates that DSA-DeepFM significantly outperforms traditional machine learning and state-of-the-art deep learning models. Furthermore, t-SNE visualizations confirm the discriminative power of the model at various stages. Additionally, we use our model to identify eight novel synergistic drug combinations, underscoring its practical utility and potential for future applications.

**Availability and implementation:**

Source code is available at https://github.com/gracygyx/DSA-DeepFM

## 1 Introduction

Compared to single-agent therapy, combination therapy utilizes multiple drugs targeting different biological pathways and mechanisms in the treatment of complex diseases such as cancer, HIV, and diabetes (Bar-On *et al.* 2018, Fisusi and Akala 2019, Jaaks *et al.* 2022). Drug combinations can improve efficacy while minimizing the toxic side effects associated often with monotherapy (Nussinov *et al.* 2013, Tong *et al.* 2017). However, inappropriate drug combinations can cause antagonistic effects or even worsen disease progression, as evidenced by a clinical study involving 48 patients diagnosed with idiosyncratic drug-induced liver injury who were taking two drugs (Benesic *et al.* 2019). Therefore, accurately designing synergistic combination regimens is essential for optimizing therapeutic outcomes in precision medicine.

The growing number of available drugs presents a significant challenge for identifying synergistic combinations through clinical experiments. Advances in high-throughput drug screening have allowed large-scale drug combination studies (Al-Lazikani *et al.* 2012), resulting in the creation of several notable databases containing synergistic and antagonistic interactions on different cell lines (O’Neil *et al.* 2016, Holbeck *et al.* 2017, Liu *et al.* 2020, Zheng *et al.* 2021). This facilitates the development of scalable AI and machine learning approaches for efficiently identifying synergistic drug combinations, complementing conventional experimental methods (Abbasi and Rousu 2024, Besharatifard and Vafaee 2024). In particular, recent advances in deep learning have significantly improved the prediction of drug combination effects (Sun *et al.* 2020, Jiang *et al.* 2020, Kuru *et al.* 2022, Liu *et al.* 2022, Wang *et al.* 2022a,b, Zhang *et al.* 2022, Torkamannia *et al.* 2023, Monem *et al.* 2023, Xu *et al.* 2023, Bertin *et al.* 2023, Hu *et al.* 2023, Abd El-Hafeez *et al.* 2024, Alam *et al.* 2024).

Although these models effectively predict drug synergies, significant challenges remain (Abbasi and Rousu 2024, Besharatifard and Vafaee 2024). For example, some models incorporate diverse drug and cell line features to enhance performance; however, this increases computational complexity and introduces noise or irrelevant data, which can reduce overall effectiveness. Furthermore, merging data from multiple databases often results in incomplete entries, leading to under-utilization of available data. Models that incorporate pre-trained language models to improve feature representation may also struggle to capture high-order interactions between different feature types, thus limiting predictive capability.

We propose an end-to-end Dual-Stage Attention-enhanced Deep Factorization Machine (DSA-DeepFM) model for predicting synergistic drug combinations as a classification task. It integrates categorical and auxiliary numerical profiles of drugs and cell line to enrich feature representation. Unlike traditional DeepFM models used in other domains (Guo *et al.* 2017, Su *et al.* 2022), DSA-DeepFM uses a DSA mechanism to fuse profiles in embedding and field spaces, improving feature learning without increasing dimensionality. Its FM module captures importance of features and their interactions (Rendle 2010), while its Deep Neural Network (DNN) module captures higher-order interactions. A residual connection to the prediction module helps to maintain gradients during training, preventing vanishing gradients and supporting more effective learning.

## 2 Materials and methods

### 2.1 Datasets and preprocessing

The databases used in this study are summarized in Table 1.

**Table 1.** A summary of the databases used in this study.

Database	Type	Source
DrugCombDB	Drug combination	http://drugcombdb.denglab.org/
O’Neil	Drug combination	PMID: 26983881
MSigDB	KEGG pathways	http://www.broadinstitute.org/msigdb/
STITCH	Drug targets	http://stitch.embl.de/
CellMinerCDB	Gene expression	https://discover.nci.nih.gov/cellminercdb/

#### 2.1.1 Drug combination datasets

DrugCombDB (accessed in February 2023; Liu *et al.* 2020) includes 448 555 drug combinations involving 2887 specific drugs and 124 human cancer cell lines across 14 tissue types. This database integrates data from the NCI-ALMANAC project (Holbeck *et al.* 2017), the AstraZeneca–Sanger dataset (Menden *et al.* 2019), and other sources.

The O’Neil (also called Merck) dataset comprises 22 737 results from experiments across 39 cancer cell lines and 38 anticancer drugs (accessed in Feb 2023; O’Neil *et al.* 2016).

#### 2.1.2 Drug molecular fingerprints

The Simplified Molecular Input Line Entry System (SMILES) representations of drugs were downloaded from the DrugCombDB database.

#### 2.1.3 KEGG pathways

About 186 KEGG pathways, involving 5244 genes, were downloaded from the Molecular Signatures Database (MSigDB) (C2 collection, accessed in February 2023; Liberzon *et al.* 2015).

#### 2.1.4 Drug target data

We downloaded the drug target data from the Search Tool for Interactions of Chemicals (STITCH) database (Szklarczyk *et al.* 2016). This database aggregates drug-protein interactions from multiple databases and literature sources.

#### 2.1.5 Cell line profiles

RNA-Seq gene expression data for the cell lines in DrugCombDB were obtained from the CellMiner Cross Database (CellMinerCDB) (Luna *et al.* 2021). This platform integrates multiple cancer cell line datasets, including NCI-60, the Broad Cancer Cell Line Encyclopedia, and Sanger/MGH Genomics of Drug Sensitivity in Cancer.

#### 2.1.6 Data preprocessing

Following the study of Preuer *et al.* (2018), we labeled drug combinations with synergy scores >30 as positive (synergistic), and those with scores below 0 as negative (antagonistic) for the O’Neil dataset.

In cases where a drug combination has multiple entries with varying synergy outcomes across the two databases, majority voting was employed to determine the overall synergistic property of the combination. After preprocessing, the DrugCombDB dataset contained 48 985 positive and 112 106 negative pairs (approximately a ratio of 1:2.3), whereas the O’Neil dataset contained 13 596 positive and 15 378 negative pairs (approximately a ratio of 1:1.1).

We converted the SMILES representations of drugs into the 1024-bit extended connectivity fingerprints with a diameter of 6 (ECFP6) using the Morgan algorithm in RDKit (Landrum *et al.* 2013). The Tanimoto coefficient was then calculated based on the ECFP6 fingerprints.

To standardize the RNA-sequencing gene expression data from CellMinerCDB, we applied z-score normalization.

### 2.2 Framework of DSA-DeepFM

**Figure 1.**
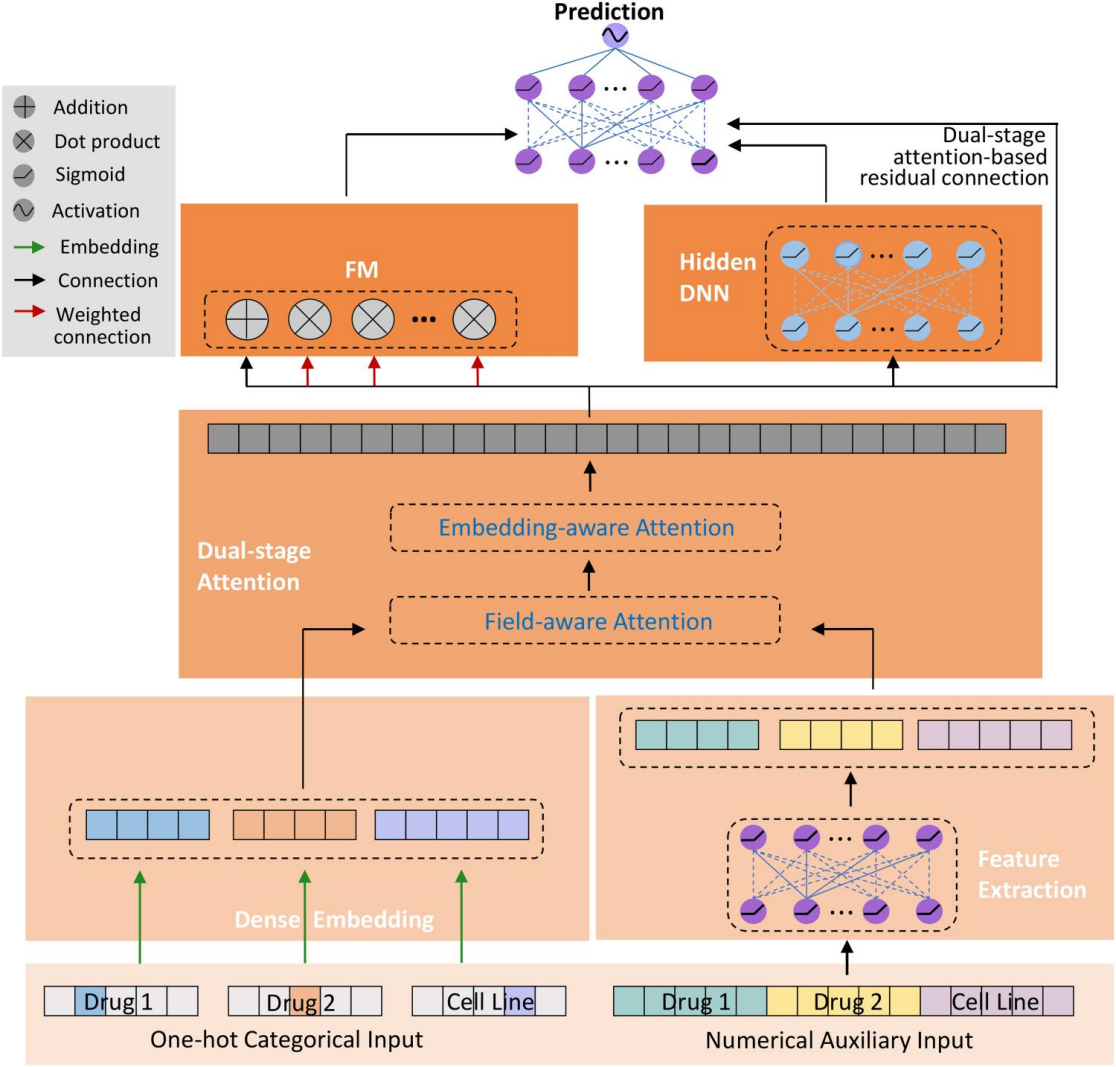
The architecture of DSA-DeepFM. The input consists of two types of data: Drug and cell line names (one-hot encoded) and auxiliary features such as drug molecular fingerprints (FPs) and cell line gene expression (GE) data. The dual-stage attention module allows both feature types to learn field-shared and embedding-space-shared representations while preserving their unique characteristics. The concatenated vector is then fed into both the FM layer and the hidden DNN module. FM captures low-order feature interactions, while hidden DNN models high-order interactions. Finally, the combined features are passed to the prediction module, which uses a residual structure to improve the prediction of synergistic drug combinations.

The DSA-DeepFM model (Fig. 1) consists of an initialization module that transforms categorical and numerical auxiliary features into latent representations, a dual-stage attention (DSA) module that integrates the two feature types through field-aware and embedding-aware attention, an FM module that captures low-order feature interactions, a hidden DNN module that learns high-order interactions, and a prediction module with DSA-based residual connections to improve prediction performance. Detailed mathematical descriptions of these modules are provided in Section 4 of the Supplementary Document, available as supplementary data at *Bioinformatics Advances* online.

#### 2.2.1 Initialization module

The initialization module consists of a dense embedding module for categorical inputs and a feature extraction module for numerical inputs.

**Dense embedding module.** Categorical inputs consist of two drugs and one cell line, each encoded as a one-hot vector corresponding to its identifier. These vectors are then mapped into dense embeddings through a trainable dictionary that learns representations of drugs and cell lines.

**Feature extraction module.** The numerical representation of each drug is a vector of Tanimoto coefficients computed against all other drugs using ECFP6 fingerprints, and that of each cell line is a vector of normalized RNA-seq values. The vectors of two drugs and one cell line are concatenated and projected through a two-layer fully connected network into the same space as the categorical embeddings.

#### 2.2.2 DSA module

**Figure 2.**
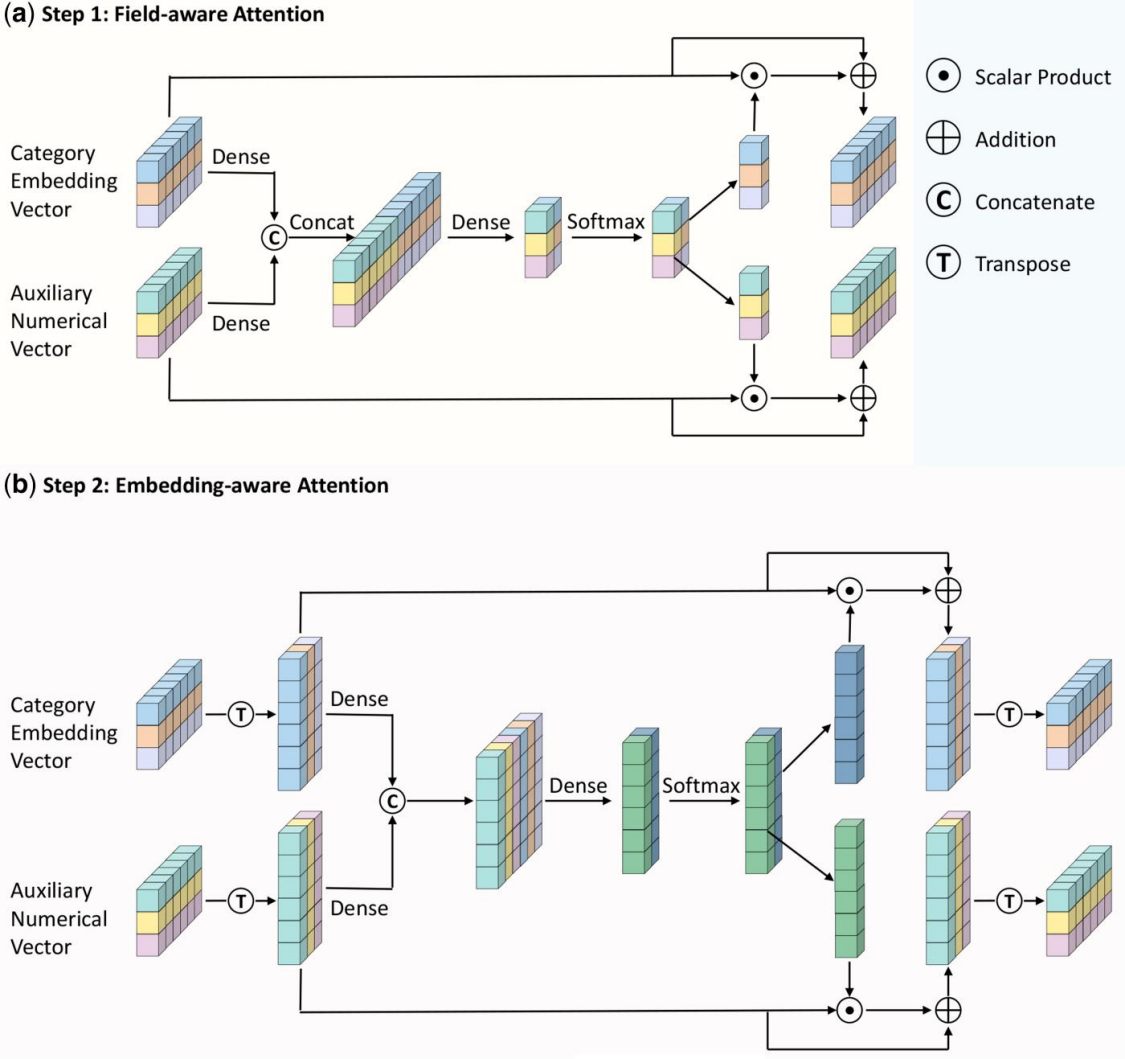
The dual-stage attention module. (a) Field-aware Attention. For each field, a two-dimensional attention vector over categorical and numerical representations is computed in a common embedding dimension space. (b) Embedding-aware Attention. For each embedding dimension, a two-dimensional attention vector over the two feature types is computed in a common field space.

To integrate categorical and numerical representations, we propose the dual-stage attention (DSA) mechanism illustrated in Fig. 2. It consists of two sequential stages. The first stage applies attention to the categorical and numerical representations at the field level, where a field denotes drug 1, drug 2, or the cell line (field-aware attention, Fig. 2a). The second stage applies attention at the embedding-dimension level in a common field space to fuse the two feature types (embedding-aware attention, Fig. 2b). This design combines latent vectors learned by the model and biologically related vectors and enables feature fusion from different perspectives.

Suppose that there are *m* drugs and *n* cell lines, the drug indices are i,j∈{1,…,m}, and the cell line indices are k∈{m+1,…,m+n}. The inputs to this module are the categorical embedding vector $x^{\text{cat}}=[x_{i}^{\text{cat}},x_{j}^{\text{cat}},x_{k}^{\text{cat}}]$ and the numerical vector $x^{\text{num}}=[x_{i}^{\text{num}},x_{j}^{\text{num}},x_{k}^{\text{num}}]$, both in $R^{3\times E}$.

**Field-aware attention module.** In this module, for each field (drug or cell line), the categorical and numerical embeddings are projected into a common space and concatenated, and a shared scoring network outputs a two-dimensional attention vector for the two feature types. The categorical embeddings $x^{\text{cat}}$ and the numerical embeddings $x^{\text{num}}$ are the first projected through separate linear transformations:


$${\hat{x}}_{i}^{\mu ,\text{field}}=x_{i}^{\mu }\cdot W^{\mu }+b^{\mu },\;\mu \in \{\text{cat},\text{num}\},$$

where $W^{\mu }\in R^{E\times E}$ and $b^{\mu }\in R^{E}$ denote the transformation parameters corresponding to each feature type. This operation maps categorical and numerical representations into a shared embedding space, enabling the model to capture field-specific features.

The transformed vectors are then concatenated and used to compute a two-dimensional attention vector for the categorical and numerical embeddings of the *i*th field:


$$\begin{array}{cc} & \left[s_{i}^{\text{cat},\text{field}},s_{i}^{\text{num},\text{field}}\right]\\ & =\text{softmax}\left(\left[{\hat{x}}_{i}^{\text{cat},\text{field}},{\hat{x}}_{i}^{\text{num},\text{field}}\right]\cdot W^{\text{field}}+b^{\text{field}}\right), \end{array}$$

where the weight matrix $W^{\text{field}}\in R^{2E\times 2}$ and bias $b^{\text{field}}\in R^{2}$. The scoring parameters $W^{\text{field}},b^{\text{field}}$ are shared across fields, establishing a common embedding-dimension space in which the importance of the two feature types within each field is assessed and subsequently used for fusion.

Finally, field-aware representations are obtained via residual connections:


$$x_{i}^{\mu ,\text{field}}=x_{i}^{\mu }\cdot s_{i}^{\mu ,\text{field}}+x_{i}^{\mu },\;\mu \in \{\text{cat},\text{num}\}.$$

Within a common embedding dimension space established by parameters shared across fields, the field aware module computes importance weights for the categorical and numerical feature types in each field and fuses them using a residual connection to obtain field specific representations and reduce redundancy.

**Embedding-aware attention module.** In this module, categorical and numerical representations are projected into a common embedding dimension space and concatenated across the three fields. A scoring network shared across embedding dimensions computes a two-dimensional attention vector, fusing the two feature types at the dimension level.

The field-aware categorical and numerical vectors $x^{\text{cat},\text{field}},x^{\text{num},\text{field}}\in R^{3\times E}$ are first transposed so that each row corresponds to an embedding dimension and each column to a field. Similar to the field-aware attention, each row vector is then passed through a linear transformation:


$${\hat{x}}_{j}^{\mu ,\text{emb}}={\left(x^{\mu ,\text{field}}\right)}_{j}^{T}\cdot W^{\mu ,\text{emb}}+b^{\mu ,\text{emb}},\;\mu \in \{\text{cat},\text{num}\}.$$

where the weight matrix $W^{\mu ,\text{emb}}\in R^{3\times 3}$ and bias $b^{\mu ,\text{emb}}\in R^{3}$.

The transformed vectors are then concatenated and used to compute the attention scores for the two feature types at the *j*th embedding dimension:


$$\begin{array}{cc} & \left[s_{j}^{\text{cat},\text{emb}},s_{j}^{\text{num},\text{emb}}\right]\\ & =\text{softmax}\left(\left[{\hat{x}}_{j}^{\text{cat},\text{emb}},{\hat{x}}_{j}^{\text{num},\text{emb}}\right]\cdot W^{\text{emb}}+b^{\text{emb}}\right), \end{array}$$

where $W^{\text{emb}}\in R^{6\times 2}$ and $b^{\text{emb}}\in R^{2}$. The parameters $W^{\text{emb}},b^{\text{emb}}$ are shared across embedding dimensions, and all computations are performed in a common field space. For each embedding dimension, the relative importance of the categorical and numerical feature types is calculated and applied to obtain a fused representation.

The embedding-aware vectors are then obtained via residual connections:


$$x_{j}^{\mu ,\text{emb}}={\left(x^{\mu ,\text{field}}\right)}_{j}^{T}\cdot s_{j}^{\mu ,\text{emb}}+{\left(x^{\mu ,\text{field}}\right)}_{j}^{T},\;\mu \in \{\text{cat},\text{num}\}.$$

With parameters shared across embedding dimensions, this module fuses categorical and numerical features at each dimension using learned importance weights and reduces feature redundancy.

In summary, the two attention stages are complementary. The field-aware stage fuses categorical and numerical information within each field, and the embedding-aware stage fuses them at the level of individual embedding dimensions, resulting in more discriminative fused representations.

Finally, the updated vectors are transposed, flattened, and concatenated to form the final feature representation:


$$x=\left[\text{flatten}\left({\left(x^{\text{cat},\text{emb}}\right)}^{T}\right),\text{flatten}\left({\left(x^{\text{num},\text{emb}}\right)}^{T}\right)\right]\in R^{6E},$$

which will be the input to both the FM and DNN modules. For clarity, we denote this dual-stage attention output by $x={\text{Attn}}_{\text{emb}}({\text{Attn}}_{\text{field}}([x^{\text{cat}},x^{\text{num}}]))$.

#### 2.2.3 FM module

The FM mechanism, originally proposed for recommender systems (Rendle 2010), captures feature relationships through the inner product of latent vectors. We use FM to evaluate feature importance and their pairwise interactions for the input vector $X=[x_{1},x_{2},\ldots ,x_{6E}]$. The output of FM is:


$$y_{\text{FM}}=x\cdot W+\sum_{i=1}^{6E}\sum_{j=i+1}^{6E}(v_{i}⊙v_{j})x_{i}x_{j},$$

where ⊙ denotes element-wise multiplication. The weight matrix $W\in R^{6E\times K}$ provides a trainable weight vector $W_{i}$ for each feature $x_{i}$, and $v_{i}\in R^{K}$ is the latent embedding vector associated with $x_{i}$. The latent dimension *K* is a hyperparameter set to 1024 in this study.

The first-order term $\sum_{i=1}^{6E}x_{i}w_{i}$ reflects the importance of individual features across the *K* dimensions. The second-order term $\sum_{i=1}^{6E}\sum_{j=i+1}^{6E}\left(v_{i}⊙v_{j}\right)x_{i}x_{j}$ models pairwise feature interactions, where $v_{i}⊙v_{j}$ captures element-wise correlations between their latent embeddings.

#### 2.2.4 Hidden DNN module

Whereas the FM module learns feature importance and pairwise interactions, the hidden DNN module captures higher-order feature interactions using the same input vector x through a two-layer neural network. The output $y_{\text{DNN}}$ is:


$$y_{\text{DNN}}=x^{\left(2\right)},\;x^{\left(l\right)}=\sigma \left(W^{\left(l\right)}x^{\left(l-1\right)}+b^{\left(l\right)}\right),\;l=1,2,$$

where $W^{\left(l\right)}$ and $b^{\left(l\right)}$ are the trainable weight and bias parameters of the *l*th layer, and σ(·) denotes the sigmoid activation function. The dimension of $y_{\text{DNN}}$ is also set to 1024, consistent with $y_{\text{FM}}$, which enables the two outputs to be directly fused in the subsequent prediction module.

#### 2.2.5 Prediction module

In the proposed architecture, the outputs of the FM and DNN modules are designed to have the same dimensionality. Before fusion, each output is normalized by batch normalization to stabilize training and reduce distributional differences. The normalized outputs are then concatenated as ${\tilde{x}}^{\left(0\right)}=[y_{\text{FM}},y_{\text{DNN}}]$, without additional feature weighting, thereby ensuring that both modules contribute equally to the fused representation. The fused vector is subsequently projected through a fully connected transformation with nonlinear activation to match the dimensionality of the DSA output x:


$${\tilde{x}}^{\left(1\right)}=\sigma \left(W^{\left(1\right)}{\tilde{x}}^{\left(0\right)}+b^{\left(1\right)}\right).$$

This formulation integrates low-order and high-order feature interactions within a unified representation space while maintaining stability and predictive performance.

We incorporate a residual connection based on the DSA mechanism, which allows the model to jointly leverage the DSA output computed before the FM branch and the DNN branch (denoted by x) and the transformed features ${\tilde{x}}^{\left(1\right)}$:


$${\tilde{x}}^{\text{attn}}={\text{Attn}}_{\text{emb}}\left({\text{Attn}}_{\text{field}}\left(\left[x,{\tilde{x}}^{\left(1\right)}\right]\right)\right).$$

Here, x denotes the output of the dual-stage attention module computed before the FM branch and the DNN branch. It concatenates the three fields from categorical and numerical data. The second input ${\tilde{x}}^{\left(1\right)}$ is the nonlinearly transformed vector produced from the outputs of the FM branch and the DNN branch and is projected to the same space as x. Attention is applied in two stages. It first operates over fields and then over embedding dimensions. ${\text{Attn}}_{\text{field}}$ and ${\text{Attn}}_{\text{emb}}$ are attention layers within the prediction module and their parameters are independent of the dual-stage attention that produces x. These two layers together form the residual fusion branch. This branch takes x together with ${\tilde{x}}^{\left(1\right)}$, which encodes interaction features of x, to mitigate overfitting. The final updated representations of x and ${\tilde{x}}^{\left(1\right)}$ are concatenated after both attention stages denoted by ${\tilde{x}}^{\text{attn}}$, and then passed to the final projection layers.

Finally, the prediction is obtained through two fully connected layers with sigmoid and softmax activations:


(1) $$y=\text{softmax}\left(\sigma \left(W^{\left(3\right)}\left(\sigma \left(W^{\left(2\right)}{\tilde{x}}^{\mathrm{attn}}+b^{\left(2\right)}\right)\right)+b^{\left(3\right)}\right)\right),$$

where *y* represents the probability that the triplet [drug 1, drug 2, cell line] exhibits a synergistic effect.

### 2.3 Experimental settings

#### 2.3.1 Validation testing

To assess our model’s performance, we compared it against nine state-of-the-art prediction methods using five-fold cross-validation, with the data split into training, validation, and test sets in a 3:1:1 ratio.

We also conduct validation tests for two more challenging leave-cell line or tissue-out cases.

- Leave-cell line-out: Cell lines in the test set were not present in the training set.
- Leave-tissue-out: The cell lines in a tissue appearing in the test set were not included in the training set.

For the leave-out scenarios, we employed the same cross-validation to compare the prediction performance of DSA-DeepFM against other models. Since O’Neil dataset is small, it is hard to design proper tests for these tests and thus we only validated the methods on DrugCombDB.

Eight metrics were used to evaluate different models: area under the receiver operating characteristic curve (AUC-ROC), area under the precision-recall curve (AUC-PR), accuracy (ACC), precision, recall, F1 score, Cohen’s Kappa (Kappa), and balanced accuracy (BACC). (For their definitions, see Section 2 of the Supplementary Document, available as supplementary data at *Bioinformatics Advances* online). Among these metrics, AUC-PR, Cohen’s Kappa, and BACC are particularly suitable for imbalanced data settings, as they better reflect performance on the minority class and mitigate bias from the majority class in the overall evaluation.

#### 2.3.2 Configuring the model’s hyperparameters

The key hyperparameters of the model were tuned based on performance on the validation datasets, measured by AUC.

**The size of the FM module.** The embedding layer of FM converts high-dimensional categorical data into dense vectors. Testing embedding dimensions from 64 to 1024 for the module showed consistent performance improvements as the dimension increased (Fig. S1.a, available as supplementary data at *Bioinformatics Advances* online). Performance stabilized at 512, with both mean and variance of AUC closely matching those at 1024. Therefore, 512 was chosen as the size of FM.

**The size of the DNN module.** The module consists of a two-layer fully connected network. Therefore, various combinations of layer dimensions were tested to optimize performance (Fig. S1.b and Section 1.1 of the Supplementary Document, available as supplementary data at *Bioinformatics Advances* online). Starting with a 256×256 network, increasing the second layer’s size improved the mean AUC and reduced variance. Further increases in the first and second layers yielded diminishing returns in performance. Given that the 512×1024 and 1024×1024 configurations produced similar results, the 512×1024 network was selected to balance performance and model complexity.

**Learning rate.** The model was trained using the Adam optimizer with learning rate decay. After tuning, a learning rate of 0.001 was selected, as it significantly improved mean AUC and reduced variance compared to 0.01, while a further decrease to 0.0001 caused a decline in performance (Fig. S1.c and Section 1.2 of the Supplementary Document, available as supplementary data at *Bioinformatics Advances* online).

**Dropout rate.** We tested dropout rates ranging from 0.0 to 0.5 to prevent overfitting. AUC increased gradually with dropout rates between 0.1 and 0.3, declined at 0.4 and then peaked at 0.5 (Fig. 1S.d and Section 1.3 of the Supplementary Document, available as supplementary data at *Bioinformatics Advances* online). Overall, dropout had minimal impact on the model’s performance. We set the drop rate to 0.5 for our model.

#### 2.3.3 Ablation analysis

The following ten variants of the DSA-DeepFM were designed to isolate the effects of different mechanisms (data types, attention, residuals) on the model’s performance:

- **DSA-DeepFM-cat**: Use only categorical data and handles inputs via embedding and feeds them into both FM and DNN components.
- **DSA-DeepFM-aux**: Processes only auxiliary data through a two-layer network before inputting it into FM and DNN.
- **DSA-DeepFM-w/o-attn**: This version processes both categorical and auxiliary data, but instead of using attention, it concatenates the vectors before inputting them into FM and DNN.
- **DSA-DeepFM-w/o-res**: In this version, residual connectivity is removed from the prediction stage, and predictions are made using a three-layer fully connected network.
- **DSA-DeepFM-attn-rev**: Here, the attention mechanism is applied in reverse order. Attention scores are first calculated in the 512-dimensional embedding space, followed by the 3-dimensional field space.
- **DSA-DeepFM-attn-field**: The attention mechanism is applied only within the three-dimensional field space, and the resulting representations are passed to the FM and DNN components.
- **DSA-DeepFM-attn-emb**: The attention mechanism is applied only in the 512-dimensional embedding space before being passed to the FM and DNN components.
- **DSA-DeepFM-attn-unified**: The dual-stage attention is replaced with a single attention layer. The categorical and numerical representations from the three fields are concatenated into a unified vector. A dense layer with sigmoid activation produces a weight vector of the same dimensionality, which is applied element-wise with a residual connection to yield the fused representation.
- **DSA-DeepFM-gated-sum**: For the fusion of FM and DNN, we replace simple concatenation with dimension-wise attention, stacking the two branch outputs, applying a shared linear layer and softmax to obtain weights at each feature dimension for FM and DNN, then combining the reweighted vectors by element-wise summation to form the fused representation.
- **DSA-DeepFM-gated-concat**: This variant is similar to DSA-DeepFM-gate-sum, except that the reweighted FM and DNN vectors are concatenated rather than summed element-wise.

## 3 Results

### 3.1 Deep learning model

The DSA-DeepFM model is illustrated in Fig. 1 and implemented using the TensorFlow backend (Version 1.14.0). The mathematical description of its components can be found in the Section 2 (and Section 4 of the Supplementary Document, available as supplementary data at *Bioinformatics Advances* online).

The input to the model consists of two types of data: the auxiliary numerical data, derived from the molecular profiles of drugs and the cell lines, and the one-hot encoded categorical data.

The categorical input is mapped to a high-dimensional feature space by the dense embedding layer, enabling the model to learn useful latent features during training. Simultaneously, a feature extraction layer processes the auxiliary information to identify significant features. These extracted features are then fused with the learned representations from the embedding layer using the DSA mechanism (Fig. 2). This integration enhances the model’s ability to classify and characterize features by effectively combining known auxiliary information with the latent representations.

Next, the embedding features and auxiliary features are concatenated into a single input vector, which is shared between the FM layer (Rendle 2010) and the Hidden DNN module. FM attempts to capture low-order feature interactions, while Hidden DNN learns high-order feature interactions. The low-order and high-order interactions are then concatenated and fed to the prediction module, along with the output from the DSA module, where residual connections based on the DSA mechanism are adopted to mitigate the vanishing gradient problem.

### 3.2 Ablation analysis

#### 3.2.1 The impact of different mechanisms on model performance

Ablation experiments were conducted by examining five different variants of DSA-DeepFM (described in the Section 2) on the DrugCombDB dataset to investigate the following questions:

- Does the use of both categorical and auxiliary data improve performance compared to using either type of data alone?
- Does incorporating an attention mechanism enhance model performance?
- How does performance differ between applying only field-aware attention, applying only embedding-aware attention, and using the dual-stage design?
- When using an attention mechanism, which architecture performs better: starting with the field space of dimension three followed by the embedding space of dimension 512, or the reverse?
- Does the dual-stage attention design outperform a single unified attention layer?
- Does attention-guided fusion of FM and DNN outputs outperform simple concatenation by reducing redundant or noisy representations and improving information efficiency?
- Does adding a residual mechanism to the prediction module improve performance?

Since the residual structure in DSA-DeepFM is based on a DSA mechanism, we excluded the residual connectivity for a fair comparison across all the models listed above. Here, we focus on AUC, as the models had similar performance across different metrics.

Table S1, available as supplementary data at *Bioinformatics Advances* online, indicates that DSA-DeepFM outperformed all variants, achieving a mean AUC of 0.982 with a standard deviation of 0.003. The details of the analysis can be found in Section 1.4 of the Supplementary Document, available as supplementary data at *Bioinformatics Advances* online.

#### 3.2.2 Sensitivity to class imbalance

To evaluate how the model performs under varying degrees of class imbalance, we adopt two strategies. First, we apply a class-weighted cross-entropy. The observed positive-to-negative ratio in the training data is about 1:2.3, so the weights for the positive and negative classes are set to 2.3 and 1. Second, we construct training sets with different positive-to-negative ratios while keeping the validation and test sets fixed. Table S2, available as supplementary data at *Bioinformatics Advances* online, summarizes the results. The model remains stable under the observed imbalance in DrugCombDB. Class weighting brings small and consistent gains on AUC-PR and BACC, while AUC-ROC is maintained. Across ratios from one to one through one to four, the class-weighted setting outperforms all resampled settings on AUC-ROC, AUC-PR, and BACC. Among the resampled ratios, one to two and one to three perform better as they retain more training data, and further downsampling leads to lower performance. Overall, the results suggest that the attention modules do not exhibit systematic bias toward the majority class and that using all available data with a class-weighted loss is preferable to downsampling.

**Table 2.** Performance of DSA-DeepFM and the nine compared models on the DrugCombDB dataset.

Model	AUC-ROC	ACC	Precision	Recall	F1	AUC-PR	Kappa	BACC
DSA-DeepFM	**0.982** ±0.003	**0.962** ±0.004	**0.943** ±0.006	**0.932** ±0.011	**0.937** ±0.008	**0.968** ±0.005	**0.910** ±0.011	**0.954** ±0.006
EN	0.763 ±0.002	0.764 ±0.004	0.745 ±0.004	0.341 ±0.019	0.468 ±0.017	0.632 ±0.006	0.342 ±0.015	0.645 ±0.008
GBM	0.805 ±0.002	0.787 ±0.001	0.755 ±0.003	0.444 ±0.002	0.560 ±0.002	0.692 ±0.003	0.431 ±0.003	0.691 ±0.001
RF	0.888 ±0.001	0.838 ±0.002	0.831 ±0.004	0.584 ±0.006	0.686 ±0.003	0.808 ±0.002	0.581 ±0.004	0.766 ±0.003
SVM	0.771 ±0.001	0.741 ±0.001	0.740 ±0.005	0.227 ±0.001	0.347 ±0.005	0.616 ±0.002	0.239 ±0.004	0.596 ±0.002
XGBoost	0.832 ±0.002	0.800 ±0.001	0.783 ±0.004	0.473 ±0.001	0.589 ±0.001	0.728 ±0.003	0.467 ±0.001	0.708 ±0.001
DeepDDS-GAT	0.846 ±0.030	0.817 ±0.023	0.781 ±0.030	0.552 ±0.072	0.646 ±0.057	0.754 ±0.049	0.528 ±0.068	0.743 ±0.036
DeepDDS-GCN	0.857 ±0.029	0.818 ±0.023	0.781 ±0.019	0.555 ±0.083	0.647 ±0.060	0.764 ±0.048	0.530 ±0.071	0.744 ±0.040
HypergraphSynergy	0.905 ±0.007	0.847 ±0.010	0.744 ±0.028	0.761 ±0.016	0.752 ±0.012	0.843 ±0.011	0.642 ±0.020	0.823 ±0.007
MatchMaker	0.886 ±0.006	0.844 ±0.003	0.789 ±0.006	0.666 ±0.013	0.722 ±0.008	0.810 ±0.007	0.615 ±0.009	0.794 ±0.005
MLP	0.858 ±0.009	0.819 ±0.009	0.723 ±0.027	0.657 ±0.016	0.688 ±0.011	0.774 ±0.014	0.560 ±0.018	0.773 ±0.007

#### 3.2.3 Drug ordering independence

We tested whether the order of drug pairs (i.e. drug A-drug B vs. drug B-drug A) affected the model’s predictions. Both combinations were included in the training and test sets during five-fold cross-validation testing using the DrugCombDB dataset. Figure S2, available as supplementary data at *Bioinformatics Advances* online, shows the predicted probabilities for all test samples, with a Pearson correlation of 0.998 between the two drug orderings. This high correlation indicates that the model’s predictions are largely independent of drug ordering. The clustering of probabilities around (0, 0) and (1, 1) shows strong discriminatory ability of DSA-DeepFM.

#### 3.2.4 Explainability: visualization of embedding vectors at different stages of DSA-DeepFM

**Figure 3.**
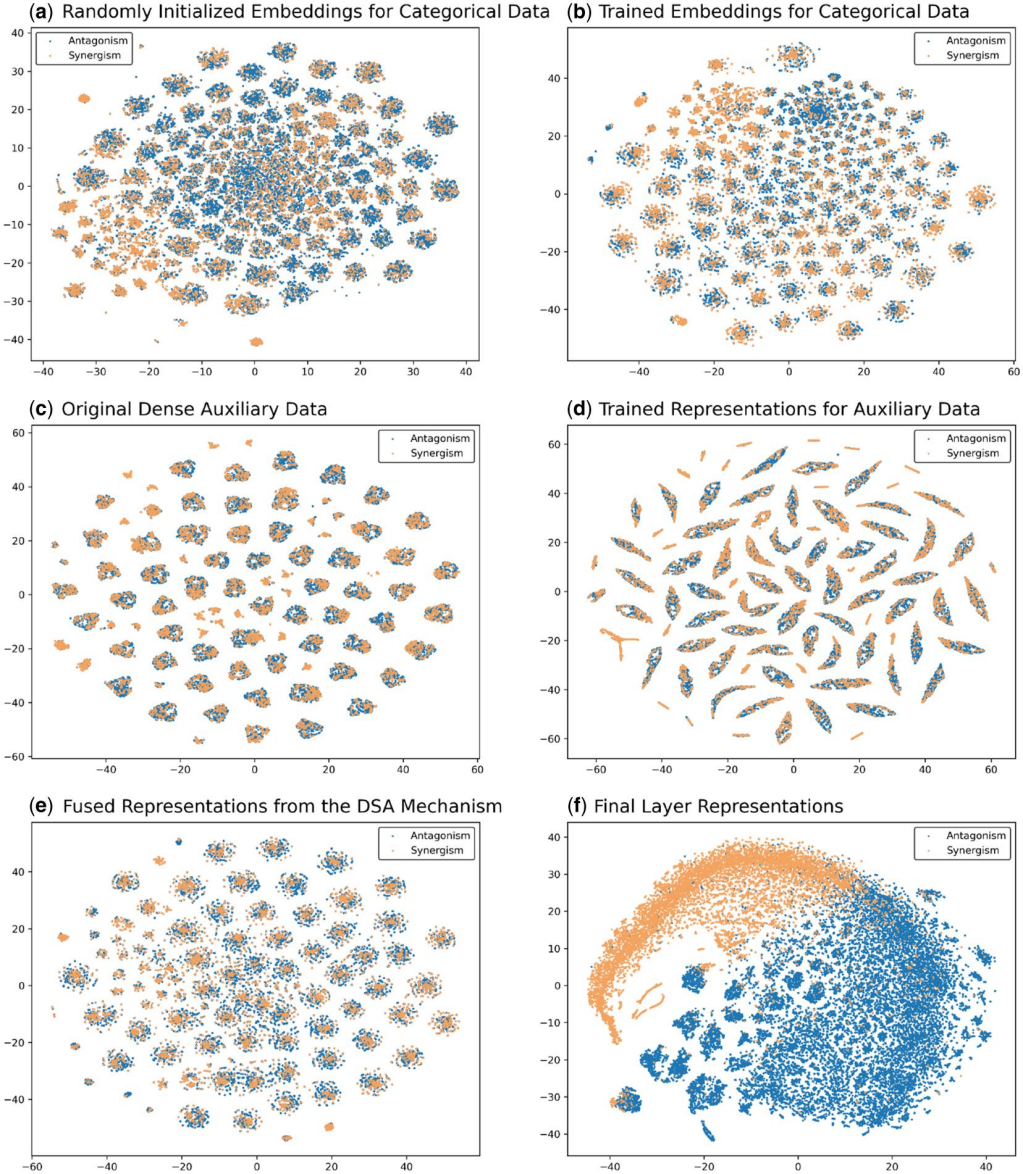
The t-SNE visualization of the representations of drug-drug-cell line triplets output at different stages of DSA-DeepFM. (a) Initialized embedding vectors from the embedding layer for categorical data before training. (b) Embedding vectors of the categorical data produced by the trained embedding layer. (c) Input auxiliary numerical data. (d) Representation vectors of the auxiliary data produced by the trained Feature Extraction module.(e) Fused representation vectors produced by the DSA mechanism that combines categorical and numerical vectors. (f) Final representation vectors used for prediction by the prediction module of the model.

We visualized the embedding features of drug-drug-cell line samples from the test set at various stages using t-distributed Stochastic Neighbor Embedding (t-SNE) to evaluate the effectiveness of DSA-DeepFM (Fig. 3, and see Section 1.5 of the Supplementary Document for details, available as supplementary data at *Bioinformatics Advances* online). Initially, the pre-training embedding layer showed no distinction between synergistic and antagonistic combinations. After training, the embedding layer improved class separation, especially for categorical data. Auxiliary data, while useful, required further processing, and the trained Feature Extraction module helped better differentiate the inputs. The fusion of categorical and auxiliary data through the attention mechanism resulted in clearer class separation, enhancing the model’s predictive power. By using the final prediction module, the model demonstrated strong classification ability, distinctly separating synergistic and antagonistic drug combinations. This analysis highlights the importance of feature fusion and the attention mechanism in improving model performance.

To strengthen the interpretation of Fig. 3, we further quantify class separation on the same test dataset using representations from each model stage, both in the original high-dimensional space and in the corresponding two-dimensional t-SNE space (Table S3, available as supplementary data at *Bioinformatics Advances* online). For each stage we applied z-score and L2 normalization and used cosine distance to compute four statistics: mean intra-class distance, inter-class centroid distance, the inter–intra margin, and the intra-to-inter ratio (definitions in Section 2.1 of the Supplementary Document, available as supplementary data at *Bioinformatics Advances* online). Compared with the untrained embeddings, the trained categorical and numerical representations show lower mean intra-class distance in the original space. From the initial to the final stage, the inter–intra margin increases from about 1.000 to about 1.274 in the t-SNE space and from about 0.342 to about 1.102 in the original space, while the intra-to-inter ratio decreases from about 0.499 to about 0.360 and from about 0.744 to about 0.382. These trends indicate tighter intra-class structure and greater inter-class separation at the model output.

**Table 3.** Performance of DSA-DeepFM and the nine compared models on the DrugCombDB dataset in leave-cell-line-out testing.

Model	AUC-ROC	ACC	Precision	Recall	F1	AUC-PR	Kappa	BACC
DSA-DeepFM	**0.817** ± **0.011**	**0.774** ± **0.010**	**0.629** ± **0.032**	**0.624** ± **0.036**	**0.626** ± **0.026**	**0.689** ± **0.031**	**0.464** ± **0.024**	**0.732** ± **0.012**
EN	0.672±0.060	0.707±0.024	0.528±0.059	0.356±0.060	0.423±0.048	0.491±0.062	0.237±0.055	0.608±0.026
GBM	0.722±0.046	0.737±0.023	0.594±0.052	0.422±0.056	0.492±0.052	0.568±0.057	0.323±0.060	0.649±0.030
RF	0.765±0.045	0.763±0.023	0.740±0.055	0.342±0.044	0.466±0.042	0.637±0.048	0.340±0.047	0.645±0.021
SVM	0.610±0.028	0.687±0.020	0.466±0.053	0.232±0.072	0.305±0.075	0.440±0.046	0.138±0.060	0.560±0.027
XGBoost	0.788±0.022	0.764±0.023	0.627±0.044	0.555±0.046	0.588±0.035	0.649±0.040	0.423±0.048	0.705±0.024
DeepDDS-GAT	0.791±0.022	0.769±0.027	0.663±0.033	0.493±0.067	0.563±0.044	0.651±0.026	0.411±0.053	0.691±0.027
DeepDDS-GCN	0.818±0.021	0.788±0.014	0.733±0.051	0.477±0.048	0.576±0.034	0.690±0.035	0.444±0.038	0.701±0.020
HypergraphSynergy	0.729±0.035	0.666±0.075	0.478±0.049	0.710±0.098	0.566±0.024	0.586±0.041	0.314±0.074	0.677±0.032
MatchMaker	0.750±0.034	0.744±0.025	0.589±0.050	0.513±0.073	0.546±0.056	0.592±0.056	0.369±0.068	0.679±0.036
MLP	0.690±0.015	0.698±0.021	0.506±0.037	0.508±0.061	0.504±0.023	0.536±0.032	0.287±0.014	0.644±0.009

### 3.3 Validation tests

Using five-fold cross-validation and leaving-cell line (or tissue)-out testing, we validated our method by comparing it with five traditional machine learning models [Random forest (RF), Support vector machine (SVM), Elastic net (EN), and Gradient Boosting machine (GBM) in the scikit-learn Python package and XGBoost in xgboost Python package] and four deep learning models [Multi-Layer Perceptron (MLP), MatchMaker (Kuru *et al.* 2022), DeepDDS (Wang *et al.* 2022a), and HypergraphSynergy (Liu *et al.* 2022)].

The average accuracy and variance of the five-fold cross-validation tests on the DrugCombDB and O’Neil datasets are summarized in Table 2 and Table S4, respectively, available as supplementary data at *Bioinformatics Advances* online. From these tables, we derive the following findings regarding the prediction of the synergistic properties of drug combinations on cell lines.

**Table 4.** Performance of DSA-DeepFM and the nine compared models on the DrugCombDB dataset in leave-tissue-out testing.

Model	AUC-ROC	ACC	Precision	Recall	F1	AUC-PR	Kappa	BACC
DSA-DeepFM	**0.804** ± **0.100**	**0.761** ± **0.073**	**0.620** ± **0.139**	**0.618** ± **0.201**	**0.585** ± **0.143**	**0.716** ± **0.124**	**0.412** ± **0.117**	**0.727** ± **0.089**
EN	0.688±0.127	0.666±0.090	0.525±0.189	0.465±0.258	0.421±0.146	0.573±0.189	0.194±0.105	0.615±0.082
GBM	0.732±0.089	0.663±0.166	0.559±0.189	0.530±0.220	0.487±0.148	0.583±0.114	0.265±0.132	0.633±0.053
RF	0.759±0.105	0.719±0.119	0.685±0.191	0.407±0.211	0.448±0.090	0.639±0.098	0.286±0.129	0.649±0.092
SVM	0.587±0.097	0.690±0.126	0.523±0.231	0.288±0.242	0.324±0.204	0.455±0.193	0.121±0.115	0.555±0.055
XGBoost	0.774±0.118	0.700±0.124	0.590±0.202	0.619±0.184	0.553±0.144	0.652±0.109	0.336±0.162	0.681±0.067
DeepDDS-GAT	0.798±0.080	0.769±0.080	0.649±0.221	0.437±0.173	0.515±0.183	0.702±0.120	0.354±0.136	0.666±0.064
DeepDDS-GCN	0.818±0.081	0.774±0.096	0.617±0.298	0.411±0.208	0.492±0.243	0.738±0.113	0.358±0.180	0.666±0.083
HypergraphSynergy	0.742±0.038	0.709±0.053	0.514±0.150	0.712±0.146	0.576±0.143	0.587±0.192	0.329±0.104	0.698±0.054
MatchMaker	0.740±0.089	0.720±0.089	0.571±0.166	0.588±0.209	0.539±0.132	0.585±0.166	0.319±0.136	0.676±0.088
MLP	0.654±0.090	0.666±0.108	0.492±0.157	0.509±0.168	0.470±0.107	0.533±0.095	0.221±0.114	0.620±0.056

- Among the five traditional machine learning methods, RF performs best on the DrugCombDB dataset, while XGBoost achieves the highest performance on the O’Neil dataset.
- HypergraphSynergy and MatchMaker are the top two compared deep learning models, with the former outperforming the latter by a small margin across all metrics except Precision.
- The accuracy improvements of our model over the other models on DrugCombDB are as least: 9% in AUC-ROC, 14% in ACC, 13% in Precision, 22% in Recall, 25% in F1, 15% in AUC-PR, 42% in Kappa, and 16% in BACC.
- The accuracy improvements of our model over the others on O’Neil dataset are at least: 5% in AUC-ROC, 14% in ACC, 14% in Precision, 10% in Recall, 14% in F1, 6% in AUC-PR, 32% in Kappa, and 13% in BACC.

In the more challenging leave-cell line-out and leave-tissue-out scenarios, we obtained mixed performance results (Tables 3 and 4). Because embeddings for unseen drugs or cell lines are untrained at evaluation, categorical embeddings were randomly initialized and kept fixed in both training and evaluation. Under this setting, our model significantly outperformed the alternatives in F1, Kappa, and BACC; however, it ranked second or third in the other five metrics.

Table S5, available as supplementary data at *Bioinformatics Advances* online, shows the leave-drug-out evaluation under the same setting. Our model ranks first in balanced accuracy, second in Kappa, third in F1, fourth in AUC-ROC and recall, fifth in ACC and AUC-PR, sixth in precision. Overall, seven of the eight metrics rank within the upper half of the 11 methods, although performance is lower than in the other leave-out scenarios. This may be due to the leave-drug-out setting, in which one or both drugs in a pair may be unseen during training, thereby increasing the difficulty of generalization.

**Table 5.** Predicted outcomes from the nine compared models on the eight drug-drug-cell line combinations given in Fig. 4, with a synergy cutoff score set at 0.6.^a^

Drug pair	Cell line	EN	GBM	RF	SVM	XGBoost	DeepDDS-GAT	DeepDDS-GCN	Hypergraph	MatchMaker	MLP
Actinomycin D + Doxorubicin	HCT116	0.505	0.642	0.739	0.470	0.905	0.090	0.466	0.502	1.000	1.000
**Etoposide + Geldanamycin**		0.613	0.673	0.703	0.497	0.955	0.003	0.097	0.431	1.000	0.967
**Etoposide + Vorinostat**		0.461	0.459	0.624	0.350	0.910	0.624	0.414	0.929	1.000	0.402
Imatinib + Mitoxantrone		0.433	0.459	0.568	0.251	0.737	0.408	0.172	0.647	0.583	0.600
Cabazitaxel + 5-Fluorouracil	SK-OV-3	0.549	0.636	0.629	0.265	0.919	0.612	0.651	0.749	0.996	1.000
Paclitaxel + Imatinib		0.328	0.364	0.423	0.251	0.731	0.304	0.336	0.461	1.000	0.993
**Paclitaxel + Vismodegib**		0.488	0.543	0.594	0.256	0.957	0.486	0.474	0.616	1.000	1.000
Paclitaxel + Axitinib		0.442	0.574	0.513	0.257	0.854	0.331	0.262	0.137	1.000	1.000

a The three combinations validated by lab experiments are highlighted in bold in the first column.

To further examine whether the model is biased toward frequent training combinations, we partitioned the test set by the minimum training frequency among drug 1, drug 2, and the cell line in each triplet and compared the highest and lowest quartiles. The high frequency quartile achieved mean AUC-ROC 0.982±0.004, the low frequency quartile achieved 0.987±0.002, and the full test set achieved 0.982±0.003. Performance is comparable across the two quartiles. The slightly higher value in the low-frequency quartile is consistent with its more balanced label distribution, with a positive class proportion ∼47% in the low-frequency quartile and ∼24% in the high-frequency quartile.

### 3.4 Discovery of novel synergistic drug combinations

**Figure 4.**
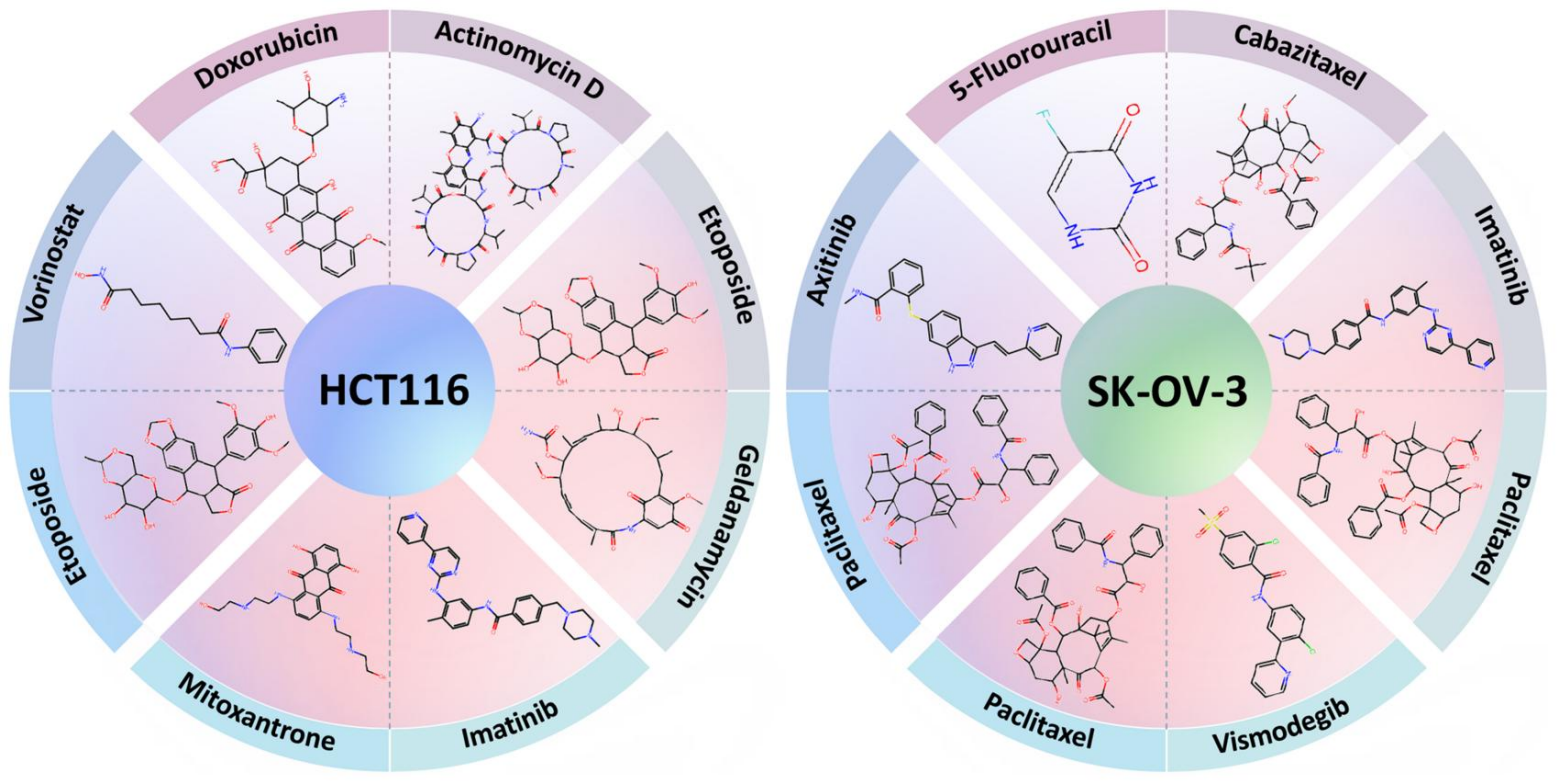
Top four predicted drug-drug combinations for the human colorectal carcinoma cell line HCT116 (left) and human ovarian cancer cell line SK-OV-3 (right).

We selected the 10 cell lines with the highest frequencies in DrugCombDB and validated all possible drug combinations that were not included in the training set. We identified the top four drug pairs in terms of likelihood for the human colorectal carcinoma cell line HCT116 and the human ovarian cancer cell line SK-OV-3 (Fig. 4). These findings are reinforced by other models (Table 5).

Etoposide and Geldanamycin combination was identified to be synergistic in HCT116. This is consistent with that targeting both Topoisomerase II and HSP90 enzymes with the predicted drug combinations accelerates and enhances apoptosis, leading to a higher percentage of apoptotic cells compared to single-drug treatments in HCT116 (Barker *et al.* 2006).

Vorinostat and Etoposide are another drug pair predicted to have a synergistic effect in HCT116. This is consistent with the observation that Vorinostat as an HDAC inhibitor induces chromatin relaxation, thereby enhancing DNA accessibility to Etoposide, which amplifies Etoposide’s ability to induce DNA damage and promote cell death Alzoubi *et al.* (2016).

Furthermore, Gray *et al.* (2012) demonstrated that Vorinostat exhibited the strongest synergistic effect when combined with either Etoposide or Topotecan in several cell lines. Although their experiments were not done on HCT116, their explanation of drug interaction mechanisms indirectly supports the synergistic effect of the Vorinostat and Etoposide combination in this cell line. Additionally, Kumar *et al.* (2018) showed that Vorinostat, in combination with Etoposide, exhibited synergistic effects in HeLa cancer cell lines.

The third predicted synergistic drug combination is Paclitaxel and Imatinib in SK-OV-3. Mundhenke *et al.* (2008) showed that combining Paclitaxel with Imatinib at concentrations of 1 or 2 µM significantly reduced cell proliferation in SK-OV-3 and three other cell lines. When the concentration of Imatinib increased to 2 or 4 µM, the reduction in proliferation became even more pronounced.

Although the remaining five predicted drug combinations lack experimental validation, the involved drug pairs have demonstrated synergistic effects in other cell lines in previous studies. Below is some indirect evidence.

- Actinomycin D exhibits anti-tumor activity, and a study on triple-negative breast cancer revealed that its combination with Doxorubicin significantly increased the apoptosis rate compared to either drug used alone (Yang *et al.* 2024).
- The combination of Mitoxantrone and Imatinib has been tested across various tumor types. Pinto *et al.* (2011) reports that it effectively reduces the required dosage of Mitoxantrone while maintaining comparable growth inhibition. Additionally, this combination is a well-tolerated treatment protocol for chronic myeloid leukemia (Fruehauf *et al.* 2007).
- A preliminary study involving 49 patients assessed the combination of axitinib and paclitaxel, administered twice daily over a 3-week treatment cycle (Kozloff *et al.* 2012). The results indicated that this combination is effective for advanced non-small cell lung cancer, further supporting the exploration of its synergistic effects in SK-OV-3.
- Lastly, research by Yeh *et al.* (2024) highlights that Vismodegib, a Hedgehog inhibitor, reduces Bax phosphorylation, thereby enhancing paclitaxel-induced cytotoxicity. This mechanism leads to mitochondrial damage and apoptosis in non-small cell lung cancer cells.

## 4 Discussion and conclusion

We have presented DSA-DeepFM for predicting the synergistic property of drug combinations. The model simultaneously processes both categorical information encoded through one-hot encoding and auxiliary numerical information on drugs and cell lines. The categorical input data leverage an embedding mechanism to extract features that are particularly relevant for classification, whereas the numerical data provide biologically useful information on drugs and cell lines.

The inclusion of the DSA mechanism enhances the model’s ability to learn shared patterns in both field and embedding spaces, resulting in features with stronger representational capabilities. The further integration of the deep FM module enables the model to capture both low- and high-order feature interactions, thereby enhancing its discriminative capability.

The model demonstrates higher accuracy and stability than existing approaches on the DrugCombDB and O’Neil datasets. The identification of eight novel drug combinations in HCT116 and SK-OV-3, partially supported by wet-lab pharmacodynamic studies reported in the literature, highlights the practical applicability of our model. Notably, none of the other deep learning models achieved the same predictions (Table 5).

However, further improvements are required to enhance the robustness of the model. One limitation is that it does not explicitly address the issue of class imbalance in the DrugCombDB and O’Neil datasets. For example, DrugCombDB contains substantially more negative than positive samples, whereas the O’Neil dataset is relatively balanced. Although DSA-DeepFM still achieves strong predictive performance under these conditions, future extensions could incorporate strategies such as sample weighting, under-sampling, or over-sampling to mitigate imbalance-related effects and further improve robustness.

Another important limitation concerns the model’s performance in patient-specific prediction scenarios. Similar to other deep learning models (Abbasi and Rousu 2024), DSA-DeepFM performs less effectively in leave-one-out cross-validation scenarios. This reduction in performance is likely due to the embedding mechanism used in the model, which may struggle with limited data in such cases. To address this, incorporating mechanisms from general models like BERT could be a promising direction. BERT’s ability to generate contextually rich embeddings might help improve the model’s performance in these more challenging validation settings.

The success of DSA-DeepFM opens up opportunities to explore its application in other areas. One promising avenue is drug response prediction (Chen and Zhang 2022, 2024), where the model’s ability to analyze multidimensional data and capture complex interactions could be leveraged to predict how cell lines or individual patients will respond to specific drug treatments. This expansion could contribute to more personalized and effective therapeutic strategies. Applications may also be possible in drug repurposing. Ongoing refinements and broader applications will be crucial for unlocking the model’s full potential in both research and clinical settings.

## Supplementary Material

vbaf269_Supplementary_Data

## Data Availability

The preprocessed DrugCombDB, O’Neil and gene datasets are available on https://drive.google.com/drive/folders/1KQU7kKH-MFl2bJhsrLv1ZHxm35Nvhhsf? usp=drive_link. Source code is available on https://github.com/gracygyx/DSA-DeepFM.
