# Supplementary material for: DSA-DeepFM: a dual-stage attention-enhanced DeepFM model for predicting anticancer synergistic drug combinations
Source: Bioinform Adv. 2025 Oct 27;5(1):vbaf269. doi: 10.1093/bioadv/vbaf269 (PMC12609172; doi:10.1093/bioadv/vbaf269)
Supplement: vbaf269_Supplementary_Data [file vbaf269_supplementary_data.zip › FigureS1.pdf]

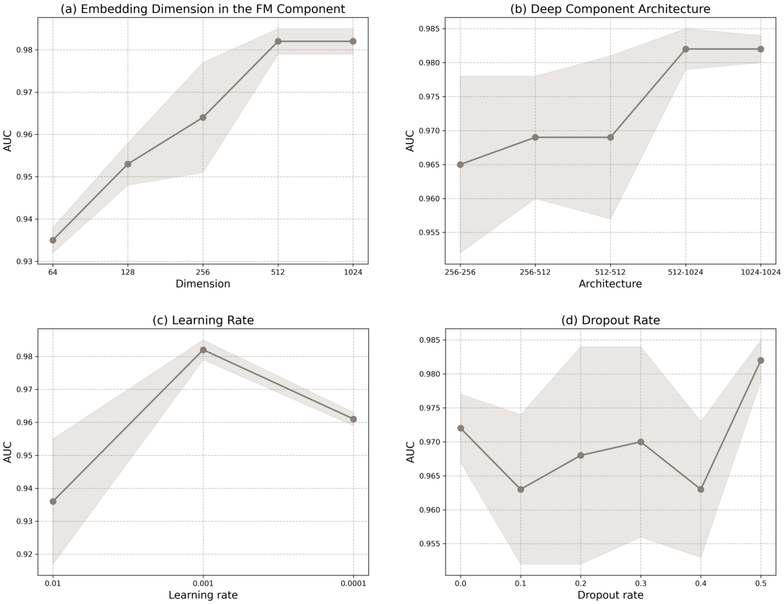

Figure S1. Parameter sensitivity analysis. (a) AUC vs. the dimension of the FM component. (b) AUC vs. the size of the deep component. (c) AUC vs. the initial learning rate during training. (d) AUC vs. the dropout rate in the hidden layers. The shaded area represents the variation of AUC.
