# Supplementary material for: DSA-DeepFM: a dual-stage attention-enhanced DeepFM model for predicting anticancer synergistic drug combinations
Source: Bioinform Adv. 2025 Oct 27;5(1):vbaf269. doi: 10.1093/bioadv/vbaf269 (PMC12609172; doi:10.1093/bioadv/vbaf269)
Supplement: vbaf269_Supplementary_Data [file vbaf269_supplementary_data.zip › Supplementary_DocumentR2.pdf]

Supplementary Materials

DSA-DeepFM: A Dual-Stage Attention-Enhanced DeepFM Model for Predicting Anticancer Synergistic Drug Combinations

Yuexi Gu, Yongheng Sun, Louxin Zhang and Jian Zu

1. Model Training

1.1 Hyperparameter settings

We examine the influence of crucial hyperparameters on model performance, with particular attention to the embedding dimension of sparse categorical data in the FM Layer, the dimensionality of the two-layer network in the DNN component, the initial learning rate and the dropout rate during model training

Embedding dimension in the FM layer
The FM layer transforms high-dimensional sparse categorical data into a dense vector. We tested the dimension of the embedding vector of 2^k for k from 6 to 10. Figure S1a shows the impact of embedding dimension on model performance in AUC. The results indicate a consistent improvement in performance as the embedding dimension increases. Notably, when the embedding dimension reaches 2^9 (i.e. 512), model's performance stabilizes, with both the mean and variance of the AUC closely aligning with those observed at an embedding dimension of 2^{10} (i.e. 1024). Consequently, we selected 512 as the optimal embedding dimension.

Dimensions of the DNN component
The DNN component consists of a two-layer fully connected network designed to capture higher-order interactions among input features. We tested the following combinations of layer dimensions: $2^8 \times 2^8$ (i.e. 256× 256), $2^8 \times 2^9$, $2^9 \times 2^9$, $2^9 \times 2^{10}$, and $2^{10} \times 2^{10}$ to determine the optimal network size. Figure 3b illustrates the effect of these dimension combinations on the model's performance, measured by AUC.

We began with a network size of $2^8 \times 2^8$. Doubling the neurons in the second layer led to an increase in the mean AUC and a reduction in variance. Next, when the neurons in the first layer were doubled, the mean AUC remained unchanged, but there was a slight increase in variance. Further doubling the neurons in the second layer resulted in a significant improvement in mean AUC and a notable decrease in variance. Finally, doubling the neurons in the first layer again left the mean AUC stable, with a slight decrease in variance, suggesting diminishing returns with further increases in network size.

To maintain a manageable number of model parameters, we decided not to expand the network size further. Given that the model's performance was nearly identical for the $2^9 \times 2^{10}$ and $2^{10} \times 2^{10}$ configurations, we ultimately adopted the $2^9 \times 2^{10}$ (i.e. 512×1024) fully connected network for the DNN component.

1.2. Learning rate

The model was trained using the Adam optimizer with learning rate decay. The initial learning rate was treated as a hyperparameter and tuned by selecting from the values 0.01, 0.001, and 0.0001. Figure S1c shows that reducing the learning rate from 0.01 to 0.001 led to a significant increase in the mean AUC and a decrease in variance. However, further decreasing the learning rate to 0.0001 resulted

in a decline in the mean AUC. Based on these observations, we selected a learning rate of 0.001 for the model.

1.3 Dropout rate

The dropout rate is a crucial hyperparameter in machine learning models, particularly for preventing overfitting during training. In our experiments, we evaluated various dropout rates, including 0 (no dropout), 0.1, 0.2, 0.3, 0.4, and 0.5, to assess their impact on model performance. As shown in Figure S1d, increasing the dropout rate from 0 to 1 resulted in a decrease in the AUC value. Dropout rates between 0.1 and 0.3 showed a gradual increase in AUC, while a rate of 0.4 led to a decline. Notably, at a dropout rate of 0.5, the mean AUC reached its highest value, with the lowest variance in AUC scores. Overall, the dropout parameter had only a minimal impact on the model's performance.

1.4 Comparison of the DSA-DeepFM and the ten alternatives

Comparing DSA-DeepFM-cat, DSA-DeepFM-aux and DSA-DeepFM-w/o-attn reveals that the best results were achieved when only categorical inputs were used, with a mean AUC-ROC of 0.897 (±0.004). This suggests that the feature fusion mechanism significantly improves the performance of a model, and the embedding layer effectively captures essential hidden features from sparse inputs.

The key difference between DSA-DeepFM-w/o-attn and DSA-DeepFM-w/o-res lies in their fusion approach. The former uses simple concatenation, while the latter employs a DSA mechanism (Figure 1). The results show that DSA-DeepFM-w/o-res significantly outperformed DSA-DeepFM-w/o-attn, with a mean AUC-ROC of 0.949 (±0.010) vs. 0.890 (±0.003). This highlights the attention mechanism's capability for the integration of different input types and enhancement of feature representation.

DSA-DeepFM-w/o-res outperformed both DSA-DeepFM-cat (mean AUC-ROC: 0.897±0.004) and DSA-DeepFM-aux (mean AUC-ROC: 0.825±0.003), demonstrating that both input types offer unique advantages for prediction, and that their combination substantially improves performance. These results highlight the importance of our proposed fusion approach and residual structure in enhancing model performance.

We also examined the influence of the sequence of the two attention mechanisms by comparing DSA-DeepFM with DSA-DeepFM-attn-rev. DSA-DeepFM again demonstrated superior performance compared to DSA-DeepFM-attn-rev, with a mean AUC-ROC of 0.982 (±0.003) versus 0.957 (±0.030). Although reversing the attention order had a less significant impact than omitting the residual structure, it still yielded considerably better results than those obtained without the residual structure or when using only a single input type.

We further compared the dual-stage attention mechanism with its single-stage variants, DSA-DeepFM-attn-field and DSA-DeepFM-attn-emb. The results demonstrated that DSA-DeepFM again achieved the best performance, with a mean AUC-ROC of 0.982 (±0.003), compared to 0.949 (±0.006) for DSA-DeepFM-attn-field and 0.970 (±0.024) for DSA-DeepFM-attn-emb. These results indicate that although a single attention stage can improve performance relative to the no-attention baseline (0.890±0.003), the dual-stage design captures more complementary information and substantially outperforms either single-stage variant.

We additionally evaluated DSA-DeepFM-attn-unified, which replaces the dual-stage attention with a single attention layer over the concatenated categorical and numerical representations from the three fields. The unified variant shows consistently lower performance than DSA-DeepFM across all metrics, with mean AUC-ROC 0.910±0.007 for DSA-DeepFM-attn-unified and 0.982±0.003 for DSA-DeepFM. Compared with the no-attention variant DSA-DeepFM-w/o-attn (mean AUC-ROC: 0.890±0.003), the improvement from a single unified layer is limited. The unified model also shows lower performance than

the single-stage variants DSA-DeepFM-attn-field (mean AUC-ROC: 0.949±0.006) and DSA-DeepFM-attn-emb (mean AUC-ROC: 0.970±0.024). Taken together, these results indicate that a two-stage design is necessary rather than a single unified attention layer.

Moreover, we evaluated two attention-guided fusion strategies between FM and DNN, DSA-DeepFM-gated-sum and DSA-DeepFM-gated-concat, against simple concatenation in DSA-DeepFM. For AUC-ROC, DSA-DeepFM-gated-concat is 0.982 ±0.002, the same mean as DSA-DeepFM (0.982 ±0.003) with a smaller standard deviation, suggesting slightly improved stability. DSA-DeepFM-gated-sum yields 0.981 ±0.002, which is slightly lower than the baseline. Overall, replacing simple concatenation with attention-guided fusion at the fusion stage of FM and DNN shows no clear AUC-ROC gain on this dataset.

1.5 Visualization of embedding vectors for drug-drug-cell line triplets

To thoroughly analyze the effectiveness of our classification model, we visualized the embedding features of drug-drug-cell line samples in the test set at various stages using t-distributed Stochastic Neighbor Embedding (t-SNE) [49]. The stages include:

- Embedding vectors produced by the embedding layer for the input categorical data before the layer was trained;
- Representation vectors from the trained embedding layer for the input categorical data;
- The input auxiliary data;
- Representation vectors from the trained two-layer network for the input auxiliary data;
- Fused representation from the trained attention mechanism, combining categorical and auxiliary vectors;
- Representation vectors produced by the trained prediction component on which prediction was made.

In Figure 3a, the scatter plot illustrates the embedded representations of the categorical inputs produced by the pre-training embedding layer. The drug-drug-cell line triplets with antagonistic effects (blue) and synergistic effects (yellow) exhibit similar distributions and are indistinguishable, reflecting the random initialization in the pre-training model.

In Figure 3b, the representations generated by the trained embedding layer for the categorical inputs are shown. Compared to the random distribution in Figure 3a, these representations demonstrate better separation between the two classes, with antagonistic samples (blue) becoming more compact. This suggests that the trained embedding layer enhances the separation between the two classes, forming distinct clusters.

Figure 3c shows the input auxiliary data. While the two types of data are somewhat distinguishable compared to Figure 3a, there remains a significant overlap between the positive and negative data entries. This indicates that the auxiliary data is useful, but requires further processing to fully realize its potential.

Figure 3d shows the representations from the auxiliary inputs produced by the trained two-layer network. Compared to the input auxiliary data in Figure 3c, the representations are more dispersed, indicating greater differentiation between data points. However, distinguishing between the two classes

remains challenging at this stage. A comparison of Figure 3c and Figure 3d shows that categorical inputs are more tightly clustered, with well-defined class centers, while numerical inputs are more scattered. This suggests that fusing both input types could enhance classification performance.

Figure 3e visualizes the representations of the fused categorical and auxiliary embeddings processed through the trained attention mechanism. Compared to Figure 3c, the data points in Figure 3e are more dispersed, with distinct class centers, whereas the class centers in Figure 3c were less pronounced. This broader dispersion of samples, along with clearer class centers, demonstrates the effectiveness of our proposed fusion approach in enhancing the model's predictive capabilities, as expected from leveraging the attention mechanism.

Figure 3f illustrates the representations from the final layer after training. At the final stage, the two classes of test samples are distinctly separated in the two-dimensional space, demonstrating the model's strong ability to predict the synergistic property.

This analysis demonstrates that the proposed fusion strategy, attention mechanism, and overall model each play a crucial role in refining feature representation and enhancing the model's classification performance. The transformation of the data points becomes increasingly effective, leading to improved accuracy in distinguishing between synergistic and antagonistic drug combinations.

2. Evaluation Metrics

2.1 Metrics for Class Separation

At each model stage, the metrics are computed on the same test set. The representations are split by the ground-truth label into a positive group $X^+ = \{\mathbf{x}_i^{(+)}\}_{i=1}^{n^+}$ of size n^+ and a negative group $X^- = \{\mathbf{x}_i^{(-)}\}_{i=1}^{n^-}$ of size n^-. To ensure comparability across stages we apply the same preprocessing at each stage, first standardizing features with z-score and then normalizing each sample to unit length using the L2 norm. Distances between samples are measured with cosine distance:

$$d_{\cos}(\mathbf{u}, \mathbf{v}) = 1 - \frac{\mathbf{u}^\top \mathbf{v}}{\|\mathbf{u}\|_2 \, \|\mathbf{v}\|_2},$$

where $\mathbf{u}$ and $\mathbf{v}$ are feature vectors. Four metrics used to assess class separation are:

- **Mean intra-class distance**: For each class $c \in \{+, -\}$, average the pairwise cosine distances among samples of that class:

$$\text{Intra}_c = \frac{1}{n_c(n_c - 1)} \sum_{\substack{i,j=1 \\ i \neq j}}^{n_c} d_{\cos}\big(\mathbf{x}_i^{(c)}, \mathbf{x}_j^{(c)}\big).$$

  The overall mean intra-class distance, weighted by class sample size, is

$$\text{Intra} = \frac{n^+ \, \text{Intra}_+ + n^- \, \text{Intra}_-}{n^+ + n^-}.$$

- **Inter-class centroid distance**: Compute the mean vector (centroid) of the positive group and of the negative group:

$$\boldsymbol{\mu}^+ = \frac{1}{n^+} \sum_{i=1}^{n^+} \mathbf{x}_i^{(+)}, \qquad \boldsymbol{\mu}^- = \frac{1}{n^-} \sum_{j=1}^{n^-} \mathbf{x}_j^{(-)}.$$

  Inter-class centroid distance is the cosine distance between class centroids:

$$\text{Inter} = d_{\cos}\left(\boldsymbol{\mu}^+, \boldsymbol{\mu}^-\right),$$

where larger values indicate greater separation between the class centers.

- **Inter–intra margin**: The difference between the inter-class centroid distance and the mean intra-class distance:

$$\text{Gap} = \text{Inter} - \text{Intra},$$

where larger values indicate better separation between classes.

- **Intra-to-inter ratio**: The ratio of the mean intra-class distance to the inter-class centroid distance:

$$\text{Ratio} = \frac{\text{Intra}}{\text{Inter}},$$

where smaller values indicate that within class distance is small relative to between class distance.

2.2 Metrics for Measuring Prediction Accuracy

We consider drug combination prediction as a binary classification problem. Define the true positive rate (TPR) to be the proportion of actual positives that are correctly identified by the model, that is

$$\text{TPR} = \frac{\text{True Positives}}{\text{True Positives} + \text{False Negatives}},$$

and define the false positive rate (FPR) as:

$$\text{FPR} = \frac{\text{False Positives}}{\text{False Positives} + \text{True Negatives}}.$$

Eight metrics are used to evaluate different models, including:

- **Area under the Receiver Operating Characteristic curve** (AUC-ROC): The ROC curve plots the TPR (y-axis) against the FPR (x-axis) at n different threshold values $\alpha_1, \alpha_2, \cdots, \alpha_n$. By adjusting the threshold for classification, the ROC curve shows the trade-offs between sensitivity (true positives) and specificity (false positives) for a binary classification problem. AUC-ROC is defined as:

$$\text{AUC-ROC} = \frac{1}{2}\sum_{i=1}^{n} (\text{FPR}_i - \text{FPR}_{i-1})(\text{TPR}_i + \text{TPR}_{i-1})$$

where TPR_i and FPR_i are the TPR and FPR at the threshold α_i.

- **Recall**: the TPR is also called the recall.

- **Precision**: It is the ratio of True Positives to the sum of True Positives and False Positives.

- **Area under the precision-recall curve** (AUC-PR):

$$\text{AUC-PR} = \frac{1}{2}\sum_{i=1}^{n} (\text{Recall}_i - \text{Recall}_{i-1})(\text{Precision}_i + \text{Precision}_{i-1})$$

where Recall_i and Precision_i are the Recall and Precision at the i-th threshold value.

- **Accuracy** (ACC): It is the ratio of correctly predicted instances to the total instances, that is

$$\text{ACC} = \frac{\text{True Positives} + \text{True Negatives}}{\text{Total Number of Instances}}$$

- **F1 score**:

$$\text{F1} = 2 \cdot \frac{\text{Precision} \cdot \text{Recall}}{\text{Precision} + \text{Recall}}$$

- **Cohen's Kappa** (Kappa):

$$\text{Kappa} = \frac{P_o - P_e}{1 - P_e}$$

  where P_o is the identical prediction rate between the two classifiers and P_e is the proportion of agreement that would be expected by chance alone, based on the distribution of categories assigned by each classifier.

- **Balanced accuracy** (BACC): It is the half of the sum of TPR and TNR.

3. Deep Learning Models Examined During Validation Testing

We compared the proposed model with the following deep learning methods during validation testing.

- MatchMaker (Kuru et al., IEEE-ACM TCBB, 2021): It comprises two parts: the drug-specific subnetworks and the synergy prediction subnetwork. The former contains two parallel sub-networks that learn the representations of each drug in a specific cell line. The latter consists of fully connected layers to predict the synergistic effects of drugs.

- DeepDDS (Wang et al., Briefings in Bioinformatics, 2022): It employs graph networks and attention mechanisms to predict the effect of drug combinations. Specifically, it uses an MLP to extract features from cell line gene expression data and uses a graph attention network or a graph convolution network to extract drug features based on molecular graphs. Finally, the embedding vectors of drug-pair-cell lines are then concatenated to predict the property of the combination. Depending on the model used for drug feature extraction, DeepDDS can be referred to as DeepDDS-GAT or DeepDDS-GCN.

- HypergraphSynergy (Liu et al., Bioinformatics,2022): It models drug-drug-cell line combinations as a hypergraph, where the nodes represent drugs and cell lines, and the hyperedges represent drug-drug-cell line interactions. By leveraging a hypergraph neural network, HypergraphSynergy generates embedding vectors for both drugs and cell lines, which are then combined to predict the synergistic properties of drug combinations.

Note that MatchMaker was originally designed to solve drug combination prediction as a regression task. To adapt them for classification, we replaced the activation function in the final layer with a sigmoid function and substituted the loss function with cross-entropy loss.

4. The Components of the DSA-DeepFM

4.1 Feature generation

Dense Embedding layer for the categorical input Assume there are m drugs and n cell lines. We use d_i^{cat} and c_j^{cat} to denote the categorical data on the i-th drug and the j-th cell line, respectively, for each i and j. For each pair of drugs and each cell line, the categorical input is the triplet $\mathbf{a}^{\text{cat}} = [d_i^{\text{cat}}, d_j^{\text{cat}}, c_k^{\text{cat}}]$. The embedding layer of our model maps each input triplet to a dense numeric vector in $\mathbb{R}^{3\times E}$ using an embedding dictionary [50, 51]. Here, each d_i^{cat} is mapped to $\mathbf{x}_i^{\text{cat}} \in \mathbb{R}^E$ and each c_j^{cat} is mapped to $\mathbf{x}_{m+j}^{\text{cat}} \in \mathbb{R}^E$. (The embedding size E was set to 512 in our study.) The

dictionary $Dict = \{\mathbf{x}_1^{\text{cat}}, \mathbf{x}_2^{\text{cat}}, ..., \mathbf{x}_{m+n}^{\text{cat}}\}$ contains the trainable latent vectors, which can be interpreted as the weights of a fully connected network and will be learned during the training process [52].

Feature Extraction module for the auxiliary feature input Let d_{ij}^{aux} be the Tanimoto coefficient between the ECFP6 fingerprints of drug d_i and d_j. For each drug d_i, its auxiliary information is represented as $\mathbf{d}_i^{\text{aux}} = [d_{i1}^{\text{aux}}, d_{i2}^{\text{aux}}, ..., d_{im}^{\text{aux}}]$. Similarly, for each k, the auxiliary information of cell line c_k is $\mathbf{C}_k^{\text{aux}} = [c_{k1}^{\text{aux}}, c_{k2}^{\text{aux}}, ..., c_{kl}^{\text{aux}}]$, where l is the number of genes used in our model, and c_{ik}^{aux} represents the normalized RNA-seq value of the k-th gene in the cell line. For each pair of drugs and each cell line, the corresponding feature input is $\mathbf{a}^{\text{aux}} = [\mathbf{d}_i^{\text{aux}}, \mathbf{d}_j^{\text{aux}}, \mathbf{C}_k^{\text{aux}}] \in \mathbb{R}^{2m+l}$.

To align the auxiliary information with the categorical embedding, we implemented a two-layer fully connected network to extract latent features from the feature input triplets, which maps each $\mathbf{a}^{\text{aux}}$ to $\mathbf{x}^{\text{aux}}$ in the same feature space $\mathbb{R}^{3\times E}$ as the categorical embedding vectors. The extracted numerical feature is denoted as $\mathbf{x}^{\text{aux}} = [\mathbf{x}_i^{\text{aux}}, \mathbf{x}_j^{\text{aux}}, \mathbf{x}_{m+k}^{\text{aux}}]$, where $\mathbf{x}_i^{\text{aux}} \in \mathbb{R}^E$.

4.2 The DSA module

To integrate the categorical embedding and numerical features, we propose a DSA mechanism (Figure 2). The inputs to this module are the categorical embedding vector $\mathbf{x}^{\text{cat}} = [\mathbf{x}_i^{\text{cat}}, \mathbf{x}_j^{\text{cat}}, \mathbf{x}_{m+k}^{\text{cat}}] \in \mathbb{R}^{3\times E}$ and the feature vector $\mathbf{x}^{\text{aux}} = [\mathbf{x}_i^{\text{aux}}, \mathbf{x}_j^{\text{aux}}, \mathbf{x}_{m+k}^{\text{aux}}] \in \mathbb{R}^{3\times E}$. The DSA module has two components: Field-aware attention (Figure 2a) and embedding-aware attention (Figure 2b).

Field-aware attention module In this stage, for each field (drug or cell line), attention is computed on the categorical and numerical embeddings in the same embedding dimension space. The categorical embedding vectors $\mathbf{x}^{\text{cat}} = [\mathbf{x}_i^{\text{cat}}, \mathbf{x}_j^{\text{cat}}, \mathbf{x}_{m+k}^{\text{cat}}]$ and the auxiliary numerical vectors $\mathbf{x}^{\text{aux}} = [\mathbf{x}_i^{\text{aux}}, \mathbf{x}_j^{\text{aux}}, \mathbf{x}_{m+k}^{\text{aux}}]$ are first updated using two different dense operations as:

$$\hat{\mathbf{x}}_i^{\text{cat,field}} = \mathbf{x}_i^{\text{cat}} \cdot \mathbf{W}^{\text{cat}} + \mathbf{b}^{\text{cat}},$$
$$\hat{\mathbf{x}}_i^{\text{aux,field}} = \mathbf{x}_i^{\text{aux}} \cdot \mathbf{W}^{\text{aux}} + \mathbf{b}^{\text{aux}},$$

where $\mathbf{W}^{\text{cat}},\ \mathbf{W}^{\text{aux}} \in \mathbb{R}^{E\times E}$ are the weight matrices, and $\mathbf{b}^{\text{cat}},\ \mathbf{b}^{\text{aux}} \in \mathbb{R}^E$ are the corresponding bias terms.

These updated vectors are concatenated into a single vector, which is then passed through another dense layer with weight matrix $\mathbf{W}^{\text{field}} \in \mathbb{R}^{2E\times 2}$ and bias $\mathbf{b}^{\text{field}} \in \mathbb{R}^2$. This operation maps the features into vectors in the same embedding space, enhancing learning shared patterns. A softmax over the two feature types is then applied to obtain a two-dimensional attention vector for the i-th field:

$$\left[s_i^{\text{cat,field}}, s_i^{\text{aux,field}}\right]$$
$$= \text{softmax}\left(\left[\hat{\mathbf{x}}_i^{\text{cat,field}}, \hat{\mathbf{x}}_i^{\text{aux,field}}\right] \cdot \mathbf{W}^{\text{field}} + \mathbf{b}^{\text{field}}\right),$$

which provides field-specific weights for the categorical and auxiliary representations. Finally, the final field-aware categorical vector $\mathbf{x}^{\text{cat,field}}$ and auxiliary vector $\mathbf{x}^{\text{aux,field}}$ are obtained via a residual connection as:

$$\mathbf{x}_i^{\text{cat,field}} = \mathbf{x}_i^{\text{cat}} \cdot s_i^{\text{cat,field}} + \mathbf{x}_i^{\text{cat}},$$
$$\mathbf{x}_i^{\text{aux,field}} = \mathbf{x}_i^{\text{aux}} \cdot s_i^{\text{aux,field}} + \mathbf{x}_i^{\text{aux}}.$$

Embedding-aware attention module In this component, attention is computed at the embedding dimension level in a common field space to fuse the categorical and numerical representations. Both the

field-aware categorical vectors $\mathbf{x}^{\text{cat,field}} = [\mathbf{x}_i^{\text{cat,field}}, \mathbf{x}_j^{\text{cat,field}}, \mathbf{x}_{m+k}^{\text{cat,field}}] \in \mathbb{R}^{3\times E}$ and field-aware auxiliary vectors $\mathbf{x}^{\text{aux,field}} = [\mathbf{x}_i^{\text{aux,field}}, \mathbf{x}_j^{\text{aux,field}}, \mathbf{x}_{m+k}^{\text{aux,field}}] \in \mathbb{R}^{3\times E}$ are first transposed so that each row now corresponds to an embedding dimension, and each column corresponds to a field.

Similar to the field-aware attention, the two vectors are passed through two dense operations and concatenated to merge their vectors into the field space. Assume the dense weight matrices are $\mathbf{W}^{\text{cat,emb}}, \mathbf{W}^{\text{aux,emb}} \in \mathbb{R}^{3\times 3}$, and assume the corresponding bias terms used are $\mathbf{b}^{\text{cat,emb}}, \mathbf{b}^{\text{aux,emb}} \in \mathbb{R}^3$. The updated categorical vectors and auxiliary vectors for the j-th embedding dimension are:

$$\hat{\mathbf{x}}_j^{\text{cat,emb}} = \left(\mathbf{x}^{\text{cat,field}}\right)_j^T \cdot \mathbf{W}^{\text{cat,emb}} + \mathbf{b}^{\text{cat,emb}},$$
$$\hat{\mathbf{x}}_j^{\text{aux,emb}} = \left(\mathbf{x}^{\text{aux,field}}\right)_j^T \cdot \mathbf{W}^{\text{aux,emb}} + \mathbf{b}^{\text{aux,emb}}.$$

These vectors $\hat{\mathbf{x}}_j^{\text{cat,emb}} \in \mathbb{R}^3$ and $\hat{\mathbf{x}}_j^{\text{aux,emb}} \in \mathbb{R}^3$ are then concatenated into a single vector and are fed to another dense layer with weight matrix $\mathbf{W}^{\text{emb}} \in \mathbb{R}^{6\times 2}$ and bias $\mathbf{b}^{\text{emb}} \in \mathbb{R}^2$. For the j-th embedding dimension, a two-dimensional attention vector for the two feature types is computed as:

$$\begin{aligned}&\left[s_j^{\text{cat,emb}}, s_j^{\text{aux,emb}}\right] \\ = \ &\text{softmax}\left(\left[\hat{\mathbf{x}}_j^{\text{cat,emb}}, \hat{\mathbf{x}}_j^{\text{aux,emb}}\right] \cdot \mathbf{W}^{\text{emb}} + \mathbf{b}^{\text{emb}}\right).\end{aligned}$$

The embedding-aware categorical vectors $\mathbf{x}^{\text{cat,emb}}$ and auxiliary vectors $\mathbf{x}^{\text{aux,emb}}$ are further updated as:

$$\mathbf{x}_i^{\text{cat,emb}} = \left(\mathbf{x}^{\text{cat,field}}\right)_i^T \cdot s_i^{\text{cat,emb}} + \left(\mathbf{x}^{\text{cat,field}}\right)_i^T,$$
$$\mathbf{x}_i^{\text{aux,emb}} = \left(\mathbf{x}^{\text{aux,field}}\right)_i^T \cdot s_i^{\text{aux,emb}} + \left(\mathbf{x}^{\text{aux,field}}\right)_i^T.$$

Finally, the vectors $(\mathbf{x}^{\text{cat,emb}})^T$ and $(\mathbf{x}^{\text{aux,emb}})^T$ are flatten and concatenated to form the final feature vector $\mathbf{X}$:

$$\begin{aligned}\mathbf{X} &= \left[\text{flatten}\left((\mathbf{x}^{\text{cat,emb}})^{\text{T}}\right), \text{flatten}\left((\mathbf{x}^{\text{aux,emb}})^{\text{T}}\right)\right] \\ &= [x_1, x_2, ..., x_{6E}].\end{aligned}$$

$\mathbf{X}$ will be the input to both the FM layer and DNN module.

4.3 FM layer

The FM mechanism, initially introduced for extracting interaction features in recommender system design [30], captures feature relationships through the inner product of two latent features. We use FM to assess both the importance of the features and the influence of their interactions for the input vector $\mathbf{X} = [x_1, x_2, ..., x_{6E}]$. The output of FM is:

$$\mathbf{y}_{FM} = W \cdot X + \sum_{i=1}^{6E} \sum_{j=i+1}^{6E} (\mathbf{V}_i \odot \mathbf{V}_j) x_i x_j$$

where $\cdot$ means matrix multiplication and $\odot$ means element-wise multiplication, $W \in \mathbb{R}^{K\times 6E}$ and $\mathbf{V}_i \in \mathbb{R}^K$ are the trainable parameters, and the hyper-parameter K was set to 1024 in this study. Considering w_i as the weight of i-th feature, we use $\sum_{i=1}^{6E} w_i x_i$ to represent the feature importance. Furthermore, we use $\langle \mathbf{V}_i, \mathbf{V}_j \rangle$ to capture the interaction between the features x_i and x_j. As a result,

$\sum_{i=1}^{6E}\sum_{j=i+1}^{6E}\langle \mathbf{V}_i, \mathbf{V}_j\rangle\, x_i x_j$ represents the overall influence of the pairwise feature interactions.

4.4 Hidden DNN module

It is the 'deep' part of our model. After the FM layer had learnt the importance of features and their pairwise interactions, DNN Hidden is used to learn the higher-order interaction among the features. This module is a two-layer Feed-Forward neural network. Its input vector is $\mathbf{X}^0 = [x_1, x_2, ..., x_{6E}]$ computed in the dual-state attention component and its output $\mathbf{y}_{DNN}$ is:

$$\mathbf{X}^{(l)} = \text{sigmoid}\left(\mathbf{W}^{(l)}\mathbf{X}^{(l-1)} + \mathbf{b}^{(l)}\right),\ \mathbf{y}_{DNN} = \mathbf{X}^{(2)},$$

where $\mathbf{W}^{(l)}$ and $\mathbf{b}^{(l)}$ are the learnable weight and bias parameters for the l-th layer.

4.5 Prediction Module

FM and Hidden DNN learn the latent features and their high-order interactions, respectively. Lastly, we concatenate these two features as the input to the prediction module. Additionally, a DSA based residual connection is added to enhance the performance of our model. The input is denoted as $\tilde{\mathbf{X}}^{(0)} = [\mathbf{y}_{FM}, \mathbf{y}_{DNN}]$. Firstly, we map $\tilde{\mathbf{X}}^{(0)}$ into the same feature space with $\mathbf{X}^{(0)}$ to align the dimension of these two vectors, resulting in:

$$\tilde{\mathbf{X}}^{(1)} = \sigma\left(\mathbf{W}^{(1)}\tilde{\mathbf{X}}^{(0)} + \mathbf{b}^{(1)}\right).$$

Next, a residual connection based on the DSA mechanism is introduced to combine $\tilde{\mathbf{X}}^{(1)}$ with the original raw feature $\mathbf{X}^{(0)}$, improving feature representation and mitigating the vanishing gradient problem.

$$\begin{aligned}
\left[\mathbf{X}_f^{(0)}, \tilde{\mathbf{X}}_f^{(1)}\right] &= \text{Attn}_{\text{field}}\left(\left[\mathbf{X}^{(0)}, \tilde{\mathbf{X}}^{(1)}\right]\right),\\
\left[\mathbf{X}_e^{(0)}, \tilde{\mathbf{X}}_e^{(1)}\right] &= \text{Attn}_{\text{emb}}\left(\left[\left(\mathbf{X}_f^{(0)}\right)^T, \left(\tilde{\mathbf{X}}_f^{(1)}\right)^T\right]\right),\\
\tilde{\mathbf{X}}^{attn} &= \left[\left(\mathbf{X}_e^{(0)}\right)^T, \left(\tilde{\mathbf{X}}_e^{(1)}\right)^T\right],\\
\tilde{\mathbf{X}}^{(2)} &= \sigma\left(\mathbf{W}^{(2)}\tilde{\mathbf{X}}^{attn} + \mathbf{b}^{(2)}\right),\\
y &= \text{softmax}\left(\sigma\left(\mathbf{W}^{(3)}\tilde{\mathbf{X}}^{(2)} + \mathbf{b}^{(3)}\right)\right),
\end{aligned}$$

where $\text{Attn}_{\text{field}}$ and Attn_{emb} denote field-aware and embedding-aware output from the DSA module, respectively. The output y represents the probability that the triplet [drug 1, drug 2, cell line] exhibits a synergistic effect.
