# Supplementary material for: DSA-DeepFM: a dual-stage attention-enhanced DeepFM model for predicting anticancer synergistic drug combinations
Source: Bioinform Adv. 2025 Oct 27;5(1):vbaf269. doi: 10.1093/bioadv/vbaf269 (PMC12609172; doi:10.1093/bioadv/vbaf269)
Supplement: vbaf269_Supplementary_Data [file vbaf269_supplementary_data.zip › TableS1-S4.pdf]

Table S1. Results of the ablation study on the DrugCombDB Dataset

Model	AUC-ROC	ACC	Precision	Recall	F1	AUC-PR	Kappa	BACC
DSA-DeepFM	**0.982**±0.003	**0.962**±0.004	**0.943**±0.006	**0.932**±0.011	**0.937**±0.008	**0.968**±0.005	**0.910**±0.011	**0.954**±0.006
DSA-DeepFM-cat	0.897±0.004	0.850±0.002	0.813±0.006	0.656±0.006	0.726±0.006	0.832±0.006	0.624±0.007	0.795±0.003
DSA-DeepFM-aux	0.825±0.003	0.798±0.002	0.749±0.006	0.504±0.006	0.602±0.004	0.718±0.004	0.474±0.005	0.715±0.003
DSA-DeepFM-w/o-attn	0.890±0.003	0.843±0.002	0.801±0.006	0.643±0.007	0.713±0.004	0.820±0.005	0.607±0.005	0.787±0.003
DSA-DeepFM-w/o-res	0.949±0.010	0.901±0.011	0.882±0.011	0.780±0.041	0.828±0.024	0.915±0.017	0.759±0.031	0.867±0.020
DSA-DeepFM-attn-rev	0.957±0.030	0.921±0.046	0.885±0.065	0.850±0.095	0.867±0.081	0.928±0.051	0.811±0.113	0.901±0.060
DSA-DeepFM-attn-field	0.949±0.006	0.896±0.007	0.856±0.005	0.790±0.020	0.821±0.013	0.910±0.008	0.748±0.018	0.866±0.010
DSA-DeepFM-attn-emb	0.970±0.024	0.943±0.038	0.914±0.059	0.894±0.073	0.904±0.066	0.948±0.042	0.863±0.093	0.929±0.048
DSA-DeepFM-attn-unified	0.910±0.007	0.870±0.002	0.814±0.006	0.744±0.004	0.777±0.004	0.860±0.006	0.686±0.005	0.835±0.003
DSA-DeepFM-gated-sum	0.981±0.002	0.961±0.005	0.943±0.010	0.927±0.008	0.935±0.008	0.968±0.004	0.907±0.011	0.951±0.005
DSA-DeepFM-gated-concat	0.982±0.002	0.960±0.004	0.940±0.007	0.928±0.007	0.934±0.007	0.969±0.004	0.905±0.009	0.951±0.005

Table S2. Robustness to class imbalance on DrugCombDB. Performance of DSA-DeepFM under different training set positive to negative ratios.

Setting	AUC-ROC	ACC	Precision	Recall	F1	AUC-PR	Kappa	BACC
Observed 1:2.3	**0.982**±0.003	**0.962**±0.004	**0.943**±0.006	**0.932**±0.011	**0.937**±0.008	**0.968**±0.005	**0.910**±0.011	**0.954**±0.006
Ratio 1:1	0.966±0.002	0.912±0.002	0.797±0.004	0.951±0.004	0.867±0.003	0.918±0.007	0.802±0.004	0.923±0.002
Ratio 1:2	0.979±0.003	0.953±0.005	0.914±0.005	0.934±0.012	0.924±0.008	0.961±0.005	0.890±0.011	0.948±0.007
Ratio 1:3	0.967±0.003	0.944±0.007	0.944±0.006	0.868±0.017	0.904±0.012	0.951±0.005	0.865±0.016	0.923±0.010
Ratio 1:4	0.947±0.013	0.918±0.019	0.936±0.024	0.783±0.047	0.853±0.038	0.923±0.022	0.797±0.050	0.880±0.027
Class-weighted	0.982±0.002	0.962±0.004	0.928±0.009	0.948±0.004	0.938±0.006	0.969±0.003	0.910±0.009	0.958±0.004

Table S3. Class separability across stages in the original feature space and t-SNE embedding

Stage	Original feature space				t-SNE embedding			
	Mean intra-class	Inter-class centroid	Inter-intra margin	Intra-to-inter ratio	Mean intra-class	Inter-class centroid	Inter-intra margin	Intra-to-inter ratio
Initial (categorical)	0.999	1.996	0.997	0.501	0.992	1.997	1.004	0.497
Initial (auxiliary)	0.995	1.337	0.342	0.744	0.998	1.998	1.000	0.499
Trained (categorical)	0.997	1.922	0.925	0.519	0.995	1.895	0.900	0.525
Trained (auxiliary)	0.957	0.107	-0.851	8.976	0.997	1.999	1.002	0.499
Fused representations	0.991	0.693	-0.298	1.430	0.994	1.996	1.002	0.498
Final layer	0.680	1.782	1.102	0.382	0.717	1.991	1.274	0.360

Table S4. Performance of DSA-DeepFM and the nine compared models on the O'Neil dataset. The highest average accuracy values for the traditional machine learning methods and deep learning methods are highlighted in blue and purple, respectively.

Model	AUC-ROC	ACC	Precision	Recall	F1	AUC-PR	Kappa	BACC
DSA-DeepFM	**0.9996**±0.0003	**0.995**±0.003	**0.995**±0.004	**0.994**±0.003	**0.994**±0.003	**0.9996**±0.0003	**0.989**±0.006	**0.995**±0.003
EN	0.827±0.004	0.748±0.007	0.755±0.008	0.686±0.008	0.719±0.007	0.814±0.007	0.492±0.014	0.745±0.007
GBM	0.904±0.005	0.822±0.006	0.825±0.007	0.788±0.007	0.806±0.006	0.896±0.006	0.641±0.012	0.820±0.006
RF	0.882±0.005	0.795±0.005	0.812±0.007	0.733±0.009	0.771±0.006	0.866±0.007	0.587±0.010	0.792±0.005
SVM	0.842±0.005	0.761±0.006	0.780±0.008	0.685±0.007	0.729±0.007	0.830±0.007	0.517±0.013	0.757±0.006
XGBoost	0.947±0.003	0.875±0.004	0.872±0.004	0.859±0.007	0.866±0.005	0.940±0.004	0.748±0.009	0.874±0.004
DeepDDS-GAT	0.868±0.005	0.788±0.004	0.777±0.006	0.769±0.005	0.773±0.004	0.848±0.007	0.575±0.009	0.787±0.004
DeepDDS-GCN	0.870±0.004	0.789±0.002	0.771±0.006	0.782±0.011	0.776±0.003	0.852±0.005	0.576±0.004	0.788±0.002
HypergraphSynergy	0.949±0.003	0.875±0.004	0.841±0.011	0.905±0.013	0.872±0.004	0.943±0.004	0.750±0.008	0.877±0.004
MatchMaker	0.928±0.003	0.851±0.003	0.845±0.007	0.836±0.008	0.840±0.003	0.922±0.004	0.701±0.006	0.850±0.003
MLP	0.910±0.008	0.835±0.011	0.807±0.013	0.851±0.022	0.828±0.013	0.880±0.017	0.669±0.023	0.836±0.012

Table S5. Performance of DSA-DeepFM and the nine compared models on the DrugCombDB dataset in Leave-Drug-Out testing. The highest average accuracy in each category is highlighted in blue or purple.

Model	AUC-ROC	ACC	Precision	Recall	F1	AUC-PR	Kappa	BACC
DSA-DeepFM	**0.712**±0.024	**0.727**±0.030	**0.595**±0.042	**0.383**±0.127	**0.455**±0.092	**0.548**±0.057	**0.283**±0.049	**0.628**±0.030
EN	0.705±0.023	0.729±0.031	0.654±0.033	0.270±0.066	0.378±0.063	0.550±0.063	0.242±0.037	0.602±0.021
GBM	0.721±0.027	0.735±0.035	0.662±0.062	0.304±0.040	0.415±0.042	0.572±0.058	0.273±0.040	0.616±0.018
RF	0.744±0.020	0.740±0.035	0.681±0.052	0.306±0.020	0.422±0.027	0.595±0.053	0.284±0.035	0.621±0.014
SVM	0.655±0.039	0.721±0.040	0.684±0.056	0.194±0.026	0.302±0.033	0.510±0.049	0.191±0.031	0.577±0.012
XGBoost	0.692±0.022	0.714±0.030	0.558±0.063	0.389±0.068	0.455±0.054	0.534±0.058	0.269±0.041	0.624±0.021
DeepDDS-GAT	0.686±0.029	0.717±0.025	0.562±0.057	0.356±0.072	0.435±0.070	0.522±0.074	0.257±0.051	0.616±0.027
DeepDDS-GCN	0.689±0.024	0.703±0.038	0.555±0.087	0.313±0.105	0.385±0.098	0.502±0.070	0.219±0.072	0.599±0.034
HypergraphSynergy	0.659±0.040	0.538±0.100	0.385±0.057	0.764±0.132	0.507±0.054	0.505±0.068	0.149±0.108	0.591±0.061
MatchMaker	0.713±0.016	0.733±0.032	0.648±0.059	0.315±0.067	0.420±0.056	0.563±0.054	0.270±0.025	0.616±0.014
MLP	0.625±0.030	0.655±0.057	0.447±0.035	0.456±0.151	0.443±0.073	0.435±0.035	0.188±0.037	0.593±0.017
