# Supplementary figures and images for: DSA-DeepFM: a dual-stage attention-enhanced DeepFM model for predicting anticancer synergistic drug combinations
Source: Bioinform Adv. 2025 Oct 27;5(1):vbaf269. doi: 10.1093/bioadv/vbaf269 (PMC12609172; doi:10.1093/bioadv/vbaf269)

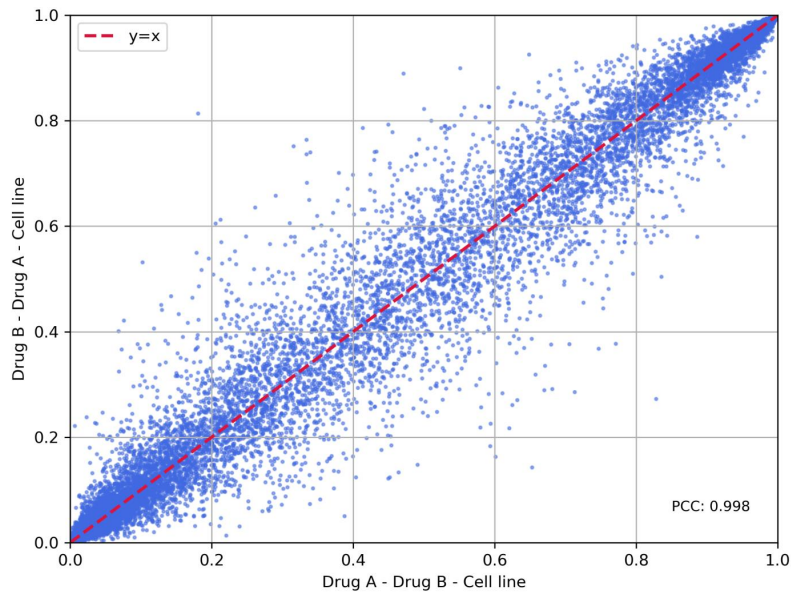

**Figure S2.** Scatter plot of predicted probabilities under different drug input orders.

Supplement: vbaf269_Supplementary_Data [file vbaf269_supplementary_data.zip › FigureS2.pdf]
